# Comparative epidemiological analysis of tumors of the digestive system in dogs and cats

**DOI:** 10.3389/fvets.2025.1701594

**Published:** 2025-11-13

**Authors:** Diana Araújo, Gabriela Fernanades da Silva, Fátima Carvalho, Nuno Vale, João Niza-Ribeiro, Ana Isabel Ribeiro, Irina Amorim, Katia Pinello

**Affiliations:** 1 Department of Pathology and Molecular Immunology, School of Medicine and Biomedical Sciences ICBAS, University of Porto, Porto, Portugal; 2 PerMed Research Group, Center for Health Technology and Services Research (CINTESIS), Porto, Portugal; 3 CINTESIS@RISE, Faculty of Medicine, University of Porto, Porto, Portugal; 4 Department of Community Medicine, Information and Health Decision Sciences (MEDCIDS), Faculty of Medicine, University of Porto, Porto, Portugal; 5 Vet-OncoNet, Population Studies Department, School of Medicine and Biomedical Sciences, ICBAS, University of Porto, Porto, Portugal; 6 EPIUnit ITR, Institute of Public Health of the University Porto, University of Porto (ISPUP), Porto, Portugal; 7 Department of Geography, Faculdade de Letras da Universidade do Porto, Centre of Studies in Geography and Spatial Planning (CEGOT), Porto, Portugal; 8 Institute of Molecular Pathology and Immunology, University of Porto (IPATIMUP), Porto, Portugal; 9 Institute for Research and Innovation in Health (i3S), University of Porto (UP), Porto, Portugal

**Keywords:** epidemiology, digestive tract neoplasms, comparative oncology, dogs, cats, Vet-OncoNet

## Abstract

**Introduction:**

Gastrointestinal (GI) disorders are a leading reason for veterinary care.

**Methods:**

This study analyzed digestive tract tumors in dogs and cats in Portugal using data from the Vet-OncoNet database, focusing on frequency, risk factors, and geographic distribution.

**Results and discussion:**

A total of 1,213 cases were included: 617 dogs (50.9%) and 596 cats (49.1%), with a higher proportion of males (54.9%) than females (45.1%). The most affected organs overall were the small intestine (26.5%) and liver/intrahepatic bile ducts (16.7%). In dogs, tumors were mainly located in the liver and bile ducts (25.8%), rectum (19.0%), small intestine (13.8%), and stomach (8.9%). In cats, the small intestine was the primary site (39.6%), followed by liver/bile ducts (7.4%), stomach (7.3%), and colon (3.5%). Lymphoma was the most common tumor type in both species (42.2%), followed by adenocarcinoma (19.0%). Among dogs, mixed breeds, Labrador Retrievers, German Shepherds, and French Bulldogs were most affected. In cats, Common European, mixed-breed, and Norwegian Forest cats predominated. The incidence rate (IR) of digestive tumors was 3.5 times higher in cats than dogs. Male cats had a 1.5 times higher IR than females. Cats also had 16 times higher risk for GI lymphoma and twice the risk for adenocarcinoma compared to dogs. Certain dog breeds, including West Highland White Terrier, Siberian Husky, and Golden Retriever, showed higher tumor incidence. Spatial analysis revealed concentration in urbanized areas, particularly around Porto and Lisbon. Conclusion: These findings highlight notable species-specific differences in digestive tract tumors, suggesting distinct genetic predispositions and possible environmental influences.

## Introduction

Gastrointestinal (GI) tract disorders are a major reason for veterinary consultation (1, 2). Although less frequent than in humans, different types of neoplasms can occur in the GI tract of domestic animals (3, 4) and in fact, spontaneous canine GI neoplasms are considered a suitable model for studying similar human tumors (5).

About 36% of all canine GI neoplasms are benign, occurring at a much higher frequency in animals (6). Various investigations report different frequencies of digestive tumors in dogs. In the United Kingdom, a study in insured dogs found a standardized incidence rate of 210 per 100,000 dogs for alimentary tumors, which accounted for 8% of all tumor cases (7). Another investigation focusing on canine GI neoplasms identified these tumors in only 1.1% of all necropsies examined (8). In Denmark, a study estimated that 5% of neoplasms in dogs involve the GI system, including the oral cavity (9). Data from the Swiss Canine Cancer Registry indicated that 7.5% of tumors diagnosed through several methods were located in the GI tract (10). In contrast, a study based on canine Japanese population reported GI tumors as the second most common neoplasms, accounting for 18.4% (including the areas from oral cavity to the anus) (11).

In cats, neoplasms of the alimentary tract account for 19.7–37.2% of all feline tumors (12). Although clinically significant, comprehensive pathological studies involving significant sample sizes are scarce (13). A retrospective study analyzed feline tumor records from Switzerland and described the GI tract as the fifth most common tumor location in this species (7.5%) with a malignancy rate of 87.1% (14).

Regarding gastric neoplasia, it accounts for less than 1% of all canine and feline neoplasms (6, 15, 16). The etiology is still unknown but long-term nitrosamine administration and even genetic factors such as breed predisposition have been associated to GC development (17). Benign tumors usually include adenomas, leiomyomas or hamartomas (15). Adenocarcinoma remains the most frequent entity in dogs, corresponding to 50–90% of all canine gastric malignancies (6, 8, 16, 18–20). Other canine gastric malignancies described comprise fibrosarcoma, leiomyosarcoma, lymphoma, mast cell tumor and extramedullary plasmacytoma (4, 15, 16).

In cats, gastric adenocarcinoma is rare, with lymphoma being the most common malignancy in this species (15, 16).

Adenomas or polyps are the most frequent benign neoplasms in canine intestinal tract (3). Neoplasms of the small intestine represents less than 1% of all malignant tumors, with the jejunum, ileum and caecum being the most common sites of occurrence (21). The most frequently reported malignancies include adenocarcinoma, lymphoma and gastrointestinal stromal tumors (GIST) (3, 21–23). In cats, intestinal tumors account for 19.2–34.8% of all alimentary tumors (12). Remarkably, intestinal lymphoma corresponds to 30% of all feline neoplasms (3, 21–23), mainly involving jejunum and ileum (21), followed by adenocarcinoma and mast cell tumors (3, 22, 23). Additionally, other tumor types can occur, such as leiomyoma, leiomyosarcoma, fibrosarcoma, hemangiosarcoma and plasma cell tumors (3, 21–23).

Despite the rarity of GI tract tumors in both canine and feline species, the great majority arise in the animal’s colon or rectum, with tumors of the large intestine representing 36–60% and 10–15% of all these neoplasms affecting dogs and cats, respectively. Among the histological tumor types are epithelial, mesenchymal, neuroendocrine and round cell. In dogs, carcinoma is the most frequent neoplasm affecting colorectal region (24).

Primary hepatic tumors are rare, accounting for approximately 0.6–1.5% and 1.0–2.9% of all canine and feline tumors, respectively (25–27). While hepatocellular adenoma is more commonly found in cats (25), hepatocellular carcinoma is the most frequent primary hepatic tumor in dogs. In cats, however, it ranks as the second most common hepatic neoplasm (26, 27). On the other hand, bile duct adenomas are benign and represent the most frequent hepatobiliary tumor in cats, accounting for 50% of all feline hepatobiliary neoplasms. Bile duct carcinomas (cholangiocarcinomas) are malignant tumors, being the most frequent malignant hepatobiliary tumor in cats and the second most common in dogs (25–27). Sarcomas of the liver are considered rare, accounting for less than 15% of all primary hepatic tumors. Among these, hemangiosarcoma is the most common type in dogs (26).

Since there are no investigations conducted in Portugal concerning this topic, the present investigation aims: (1) to describe the frequency of digestive tract tumors in dogs and cats in this geographical location; (2), to provide epidemiological indicators based on population data; (3) to analyze putative risk factors such as age, sex and breed, and (4) to assess the spatial distribution to determine whether environmental factors might be involved.

## Materials and methods

### Study design and data collection

A cross-sectional retrospective study focusing on the distribution and incidence rate (IR) of canine and feline GI tumors occurrence in Portugal was conducted. For that, data comprising confirmed diagnosis of primary canine and feline digestive tract tumors referred to Vet-OncoNet between January 2020 and December 2023 by eight veterinary laboratory partners based in Portugal (DNATECH, VetPat®, Laboratory of Pathological Anatomy from the Faculty of Veterinary Medicine of the University of Lisbon, the Laboratory of Veterinary Pathology of the University of Porto, the Laboratory of Veterinary Pathology at the University of Évora, SEGALAB, URANOLab and Cedivet) was analyzed.

The data included species, age, sex, breed, diagnosis (morphology) and anatomical location (topography), and the municipality of the requesting veterinary clinic/hospital.

Each record corresponds to a primary tumor that was classified in the Vet-OncoNet platform accordingly to the anatomical location and histological type, using the Vet-ICD-O-canine-1 (VET-ICD-O) classification system (28).

To streamline the morphological analysis, four main groups were established: (1) Adenomas, (2) Adenocarcinomas, (3) Sarcomas, and (4) Lymphomas. Supplementary Table S1 details the specific subtypes included within each group. Morphologies that did not fit into any of these categories were analyzed separately. Cases lacking a morphological diagnosis were excluded from the study. Pre-neoplastic lesions (polyps) and referred metastases were also not included.

Regarding topography, all tumors classified in the Vet-OncoNet database as “Tumors in the Digestive System and Peritoneum” (29) were included. This category encompasses topographic codes ranging from C15.- to C26.-, as well as C48.-. Cases with undefined topography, where the specific digestive organ affected could not be determined, were categorized as “Other and Ill-Defined Digestive Organs” (C26).

Regarding cat breeds, data mentioned as Common European and no breed cats were categorized as “Common European.”

### Data analysis

After completing internal validity checks and data cleaning in Microsoft Excel 2013, data analyses were performed using both Excel and R (version 4.1.2) softwares. Continuous variables were summarized as means and standard deviations, while categorical variables were presented as counts and percentages. Mean age differences were assessed using Student’s T-test and ANOVA, followed by Tukey’s *post hoc* test.

The median annual IR was calculated by summing all reported cases over the four-year study period. In order to obtain the annual average number of cases, the total was divided by four, and then divided by the population recorded in the Companion Animal Information System (SIAC) database in 2023 (data not shown), and finally multiplied by 100,000 to express the occurrence rate per 100,000 animals.

The relative risk (RR) for each breed was calculated by comparing its incidence rate to the reference groups, namely mixed-breed dogs and Common European cats. Proportional differences among groups were then assessed using the Chi-square test, applying 95% of confidence intervals (95% CI) to evaluate the precision and statistical significance of the respective findings.

For the spatial analysis, the age-standardized incidence rate (ASIR) was calculated considering the number of cases and the corresponding population within each municipality and age category. The indirect standardization method was applied, using as referential the canine population of Portugal (registered in the SIAC, in the year of 2023). Spatial analysis was conducted in GeoDa version 1.20 and mapping in ArcGIS Pro 3.1.0. The GeoDa was employed to analyze spatial autocorrelation and to identify clusters. Global spatial autocorrelation was assessed using Moran’s Index (Moran’s I) with Empirical Bayes (EB) smoothing, while local clusters were detected using the Local Index of Spatial Autocorrelation (LISA) with EB. Firstly, an EB rate was calculated by smoothing the standardized incidence ratio (SIR) derived from the “event” variable (observed cases) and the “base” variable (expected cases), in order to provide a more stable estimation of the incidence rates of digestive tract neoplasms in dogs. The smoothed SIR results were then used for both global and local Moran’s statistics. The global Moran’s I statistic was used to measure the overall degree of clustering, considering that a global Moran’s *I* > 0 indicates a clustered pattern (i.e., similar values are found near each other), *I* = 0 indicates a random pattern, and *I* < 0, a dispersed pattern.

Finally, the LISA was calculated for each municipality in the study area in order to identify spatial clusters with similar rates of digestive tract tumors, and to discriminate whether a municipality was part of a cluster of similar values (high-high or low-low) or whether it was surrounded by dissimilar values (high-low or low-high).

High-low outliers were considered to correspond to municipalities with high rates surrounded by municipalities with low rates, while low-high outliers are those with low rates surrounded by municipalities with high rates.

All statistical analyses were conducted in R, with statistical significance set at *p* < 0.05 for two-sided tests.

This study was under the approval by the Animal Welfare Ethics Committee (ORBEA) of the School of Medicine and Biomedical Sciences – ICBAS, University of Porto (P310/2019/ORBEA).

## Results

This study included 1,213 registries, of which 617 were obtained from dogs (50.9%) and 596 from cats (49.1%) (Table 1).

**Table 1:** Descriptive analysis of digestive tract tumors in dogs and cats, registered in the Vet-OncoNet database, between 2020 and 2023.

	Total		Dogs		Cats	
	*n* (%)	Mean age (SD)	*n* (%)	Mean age (SD)	*n* (%)	Mean age (SD)
Total ^(% in line)^	1,213 (100)	10.1 (3.1)	617 (50.9)	9.7 (3.0)	596 (49.1)	10.5 (3.3)*
Sex _(*n* = 1,203)_						
Female	542 (45.1)	10.2 (3.2)	285 (46.6)	10.0 (3.0)	257 (43.5)	10.4 (3.4)
Intact	492 (40.9)	10.1 (3.2)	258 (42.2)	9.9 (3.0)	234 (39.6)	10.3 (3.3)
Spayed	50 (4.2)	10.7 (3.4)	27 (4.4)	10.3 (2.9)	23 (3.9)	11.1 (3.8)
Male	661 (54.9)	10.0 (3.2)	327 (53.4)	9.5 (3.0)^t^	334 (56.5)	10.6 (3.2)
Intact	595 (49.5)	9.9 (3.1)	314 (51.3)	9.5 (3.0)	281 (47.5)	10.5 (3.2)
Castrated	66 (5.5)	10.8 (3.2)	13 (2.1)	9.8 (2.6)	53 (9.0)	11.0 (3.3)
Topography and most frequent morphologies _(Vet-ICD-O code)_						
*Small intestine _(C17.)_*	321 (26.5)	10.3 (3.2)	85 (13.8)	9.5 (3.2)	236 (39.6)	10.6 (3.1)
Lymphomas	215 (17.7)	10.2 (3.5)	23 (3.7)	7.5 (4.3)	192 (32.2)	10.6 (3.2)
Adenocarcinomas	59 (4.9)	9.9 (2.5)	31 (5.0)	9.7 (2.4)	28 (4.7)	10.1 (2.7)
Leiomyosarcoma, NOS _(8,890/3)_	17 (1.4)	10.7 (2.8)	13 (2.1)	10.8 (1.9)	4 (0.7)	10.5 (5.2)
*Liver and intrahepatic bile ducts _(C22.)_*	203 (16.7)	10.6 (2.9)	159 (25.8)	10.8 (2.8)	44 (7.4)	9.8 (3.4)
Carcinomas	59 (4.9)	11.1 (3.1)	52 (8.4)	11.2 (2.7)	7 (1.2)	10.3 (5.8)
Adenomas	56 (4.6)	11.1 (2.5)	42 (6.8)	11.3 (2.2)	14 (2.3)	10.3 (3.3)
Lymphomas	28 (2.3)	9.1 (2.9)	15 (2.4)	9.4 (3.1)	13 (2.2)	8.8 (2.7)
Hemangiosarcoma, NOS _(9,120/3)_	26 (2.1)	11.4 (2.5)	25 (4.1)	11.4 (2.6)	1 (0.2)	11.0
*Rectum _(C21.)_*	123 (10.1)	8.4 (3.1)	117 (19.0)	8.3 (3.1)	6 (1.0)	10.7 (2.8)
Adenomas	69 (5.7)	8.0 (3.3)	67 (10.9)	8.0 (3.3)	2 (0.3)	9.0 (1.4)
Adenocarcinomas	32 (2.6)	8.5 (2.5)	31 (5.0)	8.3 (2.4)	1 (0.2)	13.0
Carcinomas	7 (0.6)	10.0 (1.3)	7 (1.1)	10.0 (1.3)	0	0
*Stomach _(C16.)_*	98 (8.1)	10.3 (3.2)	55 (8.9)	10.3 (2.6)	43 (7.3)	10.2 (3.9)
Lymphomas	39 (3.2)	10.1 (4.0)	6 (1.0)	11.6 (1.9)	33 (5.5)	9.9 (4.2)
Adenocarcinomas	31 (2.6)	10.4 (2.4)	28 (4.5)	10.3 (2.4)	3 (0.5)	11.7 (2.1)
Leiomyoma, NOS _(8,890/0)_	9 (0.7)	10.7 (3.4)	9 (1.5)	10.7 (3.4)	0	0
Carcinomas	8 (0.7)	9.4 (3.1)	6 (1.0)	8.2 (2.6)	3 (0.5)	11.3 (3.2)
Round cell tumor, NOS _(8,006.1/1)_	5 (0.4)	10.2 (2.9)	1 (0.2)	7.0	4 (0.7)	11.0 (2.7)
*Colon _(C18.)_*	58 (4.8)	10.1 (2.9)	37 (6.0)	9.9 (2.5)	21 (3.5)	10.3 (3.6)
Adenocarcinomas	26 (2.1)	10.5 (2.1)	14 (2.3)	10.3 (1.2)	12 (2.0)	10.8 (2.9)
Lymphomas	9 (0.7)	8.6 (4.2)	5 (0.8)	6.2 (0.8)	4 (0.7)	11.5 (5.1)
Sarcomas	5 (0.4)	12.8 (1.5)	4 (0.6)	13.0 (1.7)	1 (0.2)	12.0
Round cell tumor, NOS _(8,006.1/1)_	3 (0.2)	7.7 (4.0)	0	0	3 (0.5)	7.7 (4.0)
*Pancreas _(C25.)_*	35 (2.9)	10.1 (2.7)	22 (3.6)	10.1 (2.4)	13 (2.2)	10.2 (3.4)
Carcinomas	13 (1.1)	10.0 (2.8)	9 (1.5)	10.3 (3.1)	4 (0.7)	9.3 (2.1)
Adenocarcinomas	7 (0.6)	10.5 (3.5)	3 (0.5)	12.0 (1.7)	4 (0.7)	9.0 (4.6)
Insulinomas	7 (0.6)	9.9 (2.8)	5 (0.8)	8.4 (1.1)	2 (0.3)	13.5 (2.1)
*Retroperitoneum and peritoneum _(C48.)_*	28 (2.3)	9.5 (2.9)	15 (2.4)	9.9 (3.1)	13 (2.2)	9.1 (2.8)
Sarcomas	7 (0.6)	9.2 (3.3)	5 (0.8)	9.5 (4.2)	2 (0.3)	8.5 (0.7)
Hemangiosarcoma, NOS _(9,120/3)_	5 (0.4)	10.4 (1.9)	5 (0.8)	10.4 (1.9)	0	0
Lymphomas	5 (0.4)	10.6 (2.9)	0	0	5 (0.8)	10.6 (2.9)
Mast cell tumor, NOS _(9,740/1)_	2 (0.2)	7.5 (0.7)	0	0	2 (0.3)	7.5 (0.7)
Mesothelioma, benign _(9,050/0)_	2 (0.2)	7.0 (4.2)	0	0	2 (0.3)	7.0 (4.2)
Carcinomas	2 (0.2)	11.0 (1.4)	1 (0.2)	10.0	1 (0.2)	12.0
Leiomyoma, NOS _(8,890/0)_	1 (0.1)	12.0	1 (0.2)	12.0	0	0
Liposarcoma, NOS _(8,850/3)_	1 (0.1)	11.0	1 (0.2)	11.0	0	0
Neoplasm, malignant _(8,000/3)_	1 (0.1)	3.0	1 (0.2)	3.0	0	0
Round cell tumor, NOS _(8,006.1/1)_	1 (0.1)	12.0	1 (0.2)	12.0	0	0
*Gallbladder _(C23.)_*	8 (0.7)	8.6 (2.1)	5 (0.8)	7.8 (2.5)	3 (0.5)	9.7 (0.6)
Carcinomas	4 (0.3)	8.0 (2.7)	3 (0.5)	7.3 (2.9)	1 (0.2)	10.0
Adenomas	2 (0.2)	10.0	1 (0.2)	–	1 (0.2)	10.0
Adenocarcinomas	1 (0.1)	9.0	0	0	1 (0.2)	9.0
Leiomyosarcoma, NOS _(8,890/3)_	1 (0.1)	9.0	1 (0.2)	9.0	0	0
*Anus and Anal Canal _(C20.)_*	6 (0.5)	9.2 (4.8)	4 (0.6)	7.5 (4.4)	2 (0.3)	12.5 (4.9)
Adenomas	2 (0.2)	6.0 (4.2)	2 (0.3)	6.0 (4.2)	0	0
Adenocarcinomas	1 (0.1)	16.0	0	0	1 (0.2)	16.0
Carcinomas	1 (0.1)	5.0	1 (0.2)	5.0	0	0
Neoplasm, malignant _(8,000/3)_	1 (0.1)	13.0	1 (0.2)	13.0	0	0
Sarcomas	1 (0.1)	9.0	0	0	1 (0.2)	9.0
*Esophagus _(C15.)_*	3 (0.2)	11 (3.5)	2 (0.3)	13 (0.0)	1 (0.2)	7.0
Adenocarcinomas	1 (0.1)	13.0	1 (0.2)	13.0	0	0
Carcinomas	1 (0.1)	7.0	0	0	1 (0.2)	7.0
Leiomyosarcoma, NOS _(8,890/3)_	1 (0.1)	13.0	1 (0.2)	13.0	0	0
*Other and ill-defined digestive organs _(C26.)_*	330 (27.2)	10.1 (3.3)	116 (18.8)	9.3 (2.8)	214 (35.9)	10.6 (3.4)
Lymphomas	210 (17.3)	10.2 (3.4)	40 (6.5)	8.4 (3.4)	170 (28.5)	10.6 (3.3)
Adenocarcinomas	64 (5.3)	9.6 (2.9)	35 (5.7)	9.3 (2.3)	29 (4.9)	10.0 (3.6)
Leiomyosarcoma, NOS _(8,890/3)_	14 (1.2)	10.1 (2.9)	13 (2.1)	9.8 (2.8)	1 (0.2)	14.0
Sarcomas	11 (0.9)	10.1 (2.8)	7 (1.1)	9.9 (1.8)	4 (0.7)	10.7 (5.0)

Vet-ICD-O code for groups as adenocarcinomas, adenomas, carcinomas, insulinomas, lymphomas and sarcomas are mentioned in Supplementary Table S1. *p < 0.05 in t-test dogs versus cats. ^t^*p* < 0.05 in t-test female versus males.

### Age and sex

The mean age of diagnosis of digestive tumors was significantly lower in dogs (9.7 years) than in cats (10.5 years) (*p* < 0.001). Males (*n* = 661, 54.9%) represented a higher proportion of the studied population compared to females (*n* = 542, 45.1%). Intact animals were more common in both species and in both sexes (males: *n* = 595, 49.5% and females: *n* = 492, 40.9%), while spayed (*n* = 50, 4.2%) or castrated (*n* = 66, 5.5%) individuals represented a smaller percentage.

Analyzing the combined data from both species, no significant differences were observed in the mean age between sexes (females *vs.* males), between intact females and intact males, or between spayed females and castrated males. At the time of diagnosis, male dogs presented a lower mean age than females (*p* < 0.05). However, no significant age differences were observed between intact and spayed or castrated dogs. In cats, age does not differ between sexes nor between intact and spayed or castrated individuals.

### Topography

Overall, the most frequent affected digestive organ was small intestine (*n* = 321, 26.5%), followed with liver and intrahepatic bile ducts (*n* = 203, 16.7%). In dogs, the latest were the locations most often compromised (*n* = 159, 25.8%), followed by the rectum (*n* = 117, 19.0%), the small intestine (*n* = 85, 13.8%) and the stomach (*n* = 55, 8.9%). In cats, tumors were more frequently diagnosed in small intestine (*n* = 236, 39.6%), followed by liver and intrahepatic bile ducts (*n* = 44, 7.4%), stomach (*n* = 43, 7.3%), and colon (*n* = 21, 3.5%).

Neoplasms in other and ill-defined digestive organs were more commonly reported in cats (*n* = 214, 35.9%) compared to dogs (*n* = 116, 18.8%).

Analyzing the combined data from both species, the post-hoc Tukey test identified statistically significant differences in the mean age of the affected patients.

Significant differences were observed with the post-hoc Tukey test between the age of animals with rectal tumors (8.4 years, SD = 3.1) and those with tumors in the colon (10.1 years, SD = 2.9), liver (10.6 years, SD = 2.9), other/ill-defined organs (10.1 years, SD = 3.3), small intestine (10.3 years, SD = 3.2), and stomach (10.3 years, SD = 3.2).

Dogs with hepatic tumors presented a significantly higher mean age compared to those presenting lesions in rectum (*p* < 0.001), small intestine (*p* = 0.017), and other/ill-defined (*p* < 0.001). Additionally, the mean age of stomach tumors was significantly higher than that of rectal tumors (*p* = 0.048).

For cats, the post-hoc Tukey test did not reveal any statistically significant differences, indicating a relatively homogeneous age distribution across animals with different tumor locations.

When comparing mean age between dogs and cats, the t-test showed significant differences in the ages of animals with tumors in other/ill-defined organs (dogs: 9.3 years, SD = 2.8; cat: 10.6 years, SD = 3.4) (*p* < 0.001), in the ages of animals with small intestine tumors (dogs: 9.5 years, SD = 3.2; cat: 10.6 years, SD = 3.1) (*p* < 0.001), and in the ages of animals with tumors in rectum (dogs: 8.3 years, SD = 3.1; cat: 10.7 years, SD = 2.8) (*p* = 0.004).

### Morphology

Regarding morphology, lymphoma was the predominant type in both species (42.2%, *n* = 512) (Supplementary Table S1). However, in cats, they account for about 70.1% of all tumors (*n* = 418). Feline lymphomas affected predominantly the small intestine (*n* = 192, 32.2%) while in dogs, other and ill-defined digestive organs (*n* = 40, 6.5%) were most commonly involved (Table 1).

The second most frequent morphology verified was adenocarcinoma which accounted of 19.0% of all cases (*n* = 230), being Adenocarcinomas NOS the most common type in both species (dogs, *n* = 103, 16.7% and cats: *n* = 61, 10.2%) (Supplementary Table S1). Adenocarcinomas were also frequent in dogs, particularly in small intestine (*n* = 31, 5.0%), rectum (*n* = 31, 5.0%) and stomach (*n* = 28, 4.5%), whereas in cats, adenocarcinomas were less diagnosed (Table 1). The frequency of adenomas was 5.7 times higher in dogs (*n* = 120, 19.4%) than in cats (*n* = 20, 3.4) (Supplementary Table S1).

Leiomyosarcomas affected both species but more often dogs (*n* = 36, 5.8%) than cats (*n* = 4, 0.7%), (Supplementary Table S1) and were mainly located the canine small intestine (*n* = 13, 2.1%) (Table 1).

In certain tumor types, the mean age of affected individuals differs between species. Cats with small intestine lymphoma were significantly older (10.6 years, SD = 3.2) than dogs with identical morphology and topography tumors (7.5 years, SD = 4.3) (*p* < 0.001).

### Breed

**Figure 1:**
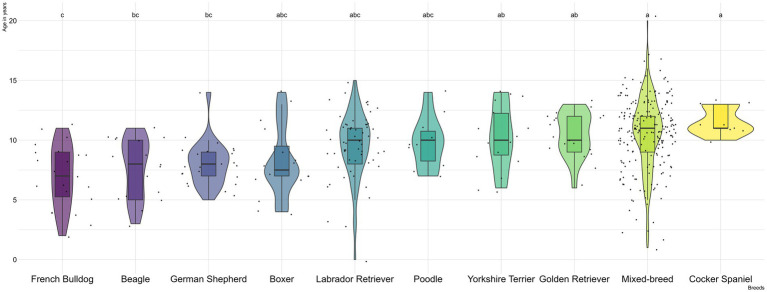
Violin plot representing the age of canine digestive tract tumors among the major breeds included in the study (with *n* > 10). The letters represent differences in the mean ages, according with ANOVA and the Tukey tests.

With respect to canine breeds, the mixed breed was the most commonly affected (*n* = 220, 35.7%), followed by Labrador Retriever (*n* = 61, 9.9%), German Shepherd (*n* = 24, 3.9%) and French Bulldog (*n* = 22, 3.6%) (Supplementary Table S2). In addition, French Bulldogs (7.0 years, SD = 2.6), Beagles (7.6 years, SD = 2.5), and German Shepherds (8.0 years, SD = 1.9) exhibited the youngest mean ages, which were significantly lower than those observed in Yorkshire Terriers (10.3 years, SD = 2.7), mixed-breed dogs (10.6 years, SD = 3.0), and Cocker Spaniels (11.6 years, SD = 1.1) (Figure 1 and Supplementary Table S2).

In cats, Common European was the predominant breed (*n* = 432, 72.5%), followed by mixed-breed (*n* = 49, 8.2%) and Norwegian Forest cat (*n* = 8, 1.3%) (Supplementary Table S3). Although no significant statistical differences between age and breed were detected amongst cats, Siamese (11.8 years, SD = 1.7) and Bengal (12.0 t years, SD = 0.0) cats developed tumors at older ages, in contrast with Norwegian Forest cats (8.0 years, SD = 3.3) which presented tumors at younger ages (Supplementary Table S3).

### Incidence rate and relative risk

**Figure 2:**
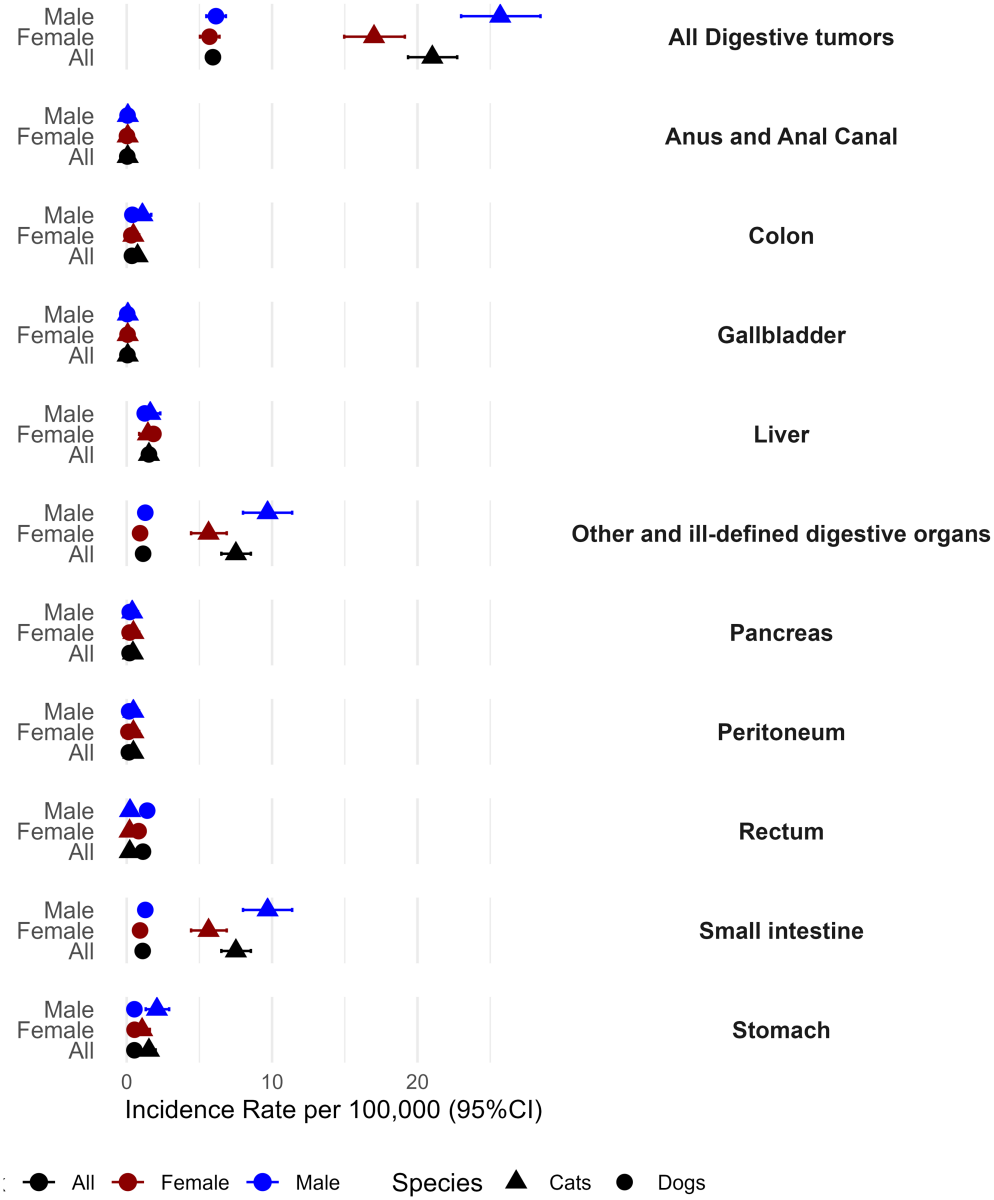
Incidence rate of digestive tract tumors, according with the different topographies and animal species.

The overall IR of digestive tumors in cats (21.0 cases per 100,000, 95% CI 19.35–22.73), was 3.5 times higher compared to dogs (5.9 cases per 100,000, 95% CI 5.46–6.40). Also, male cats (IR = 25.7, 95% CI 23.00–28.46) showed an IR 1.5 times higher than females (IR = 17.0, 95%CI 14.96–19.13) whereas in dogs, the difference of IR between sex was low (Figure 2 and Supplementary Table S4).

When analyzing the differences regarding topography (Figure 2 and Supplementary Table S4), the IR of developing tumors in “other and ill-defined” digestive organs is more than six times greater in cats (7.5, CI95% 6.51–8.54) than in dogs (1.1, CI95% 0.93–1.35). Also, cats display a two times higher IR of gastric tumors than dogs (cats 1.1 CI95% 0.60–1.59; dogs 0.5 CI95% 0.40–0.68).

In both species, males (cats 9.7 CI95% 8.00–11.38; dogs 1.3 CI95% 0.98–1.60) exhibited a higher IR for small intestine tumors, compared to females (cats 5.6 CI95% 4.43–6.88, dogs 0.9 CI95% 0.66–1.20) (Figure 2 and Supplementary Table S4). Similar findings were also observed for “other and ill-defined” digestive organ tumors.

Another interesting finding was noted with regard to the occurrence of rectal tumors for which dogs displayed a five times higher IR when compared with cats (dogs 1.1 CI95% 0.92–1.34; cats 0.2 CI95% 0.07–0.39), with male dogs exhibiting a pronounced IR (1.4 CI95% 1.11–1.73) (Figure 2 and Supplementary Table S4).

In all the other locations analyzed, both species presented similar IR.

**Figure 3:**
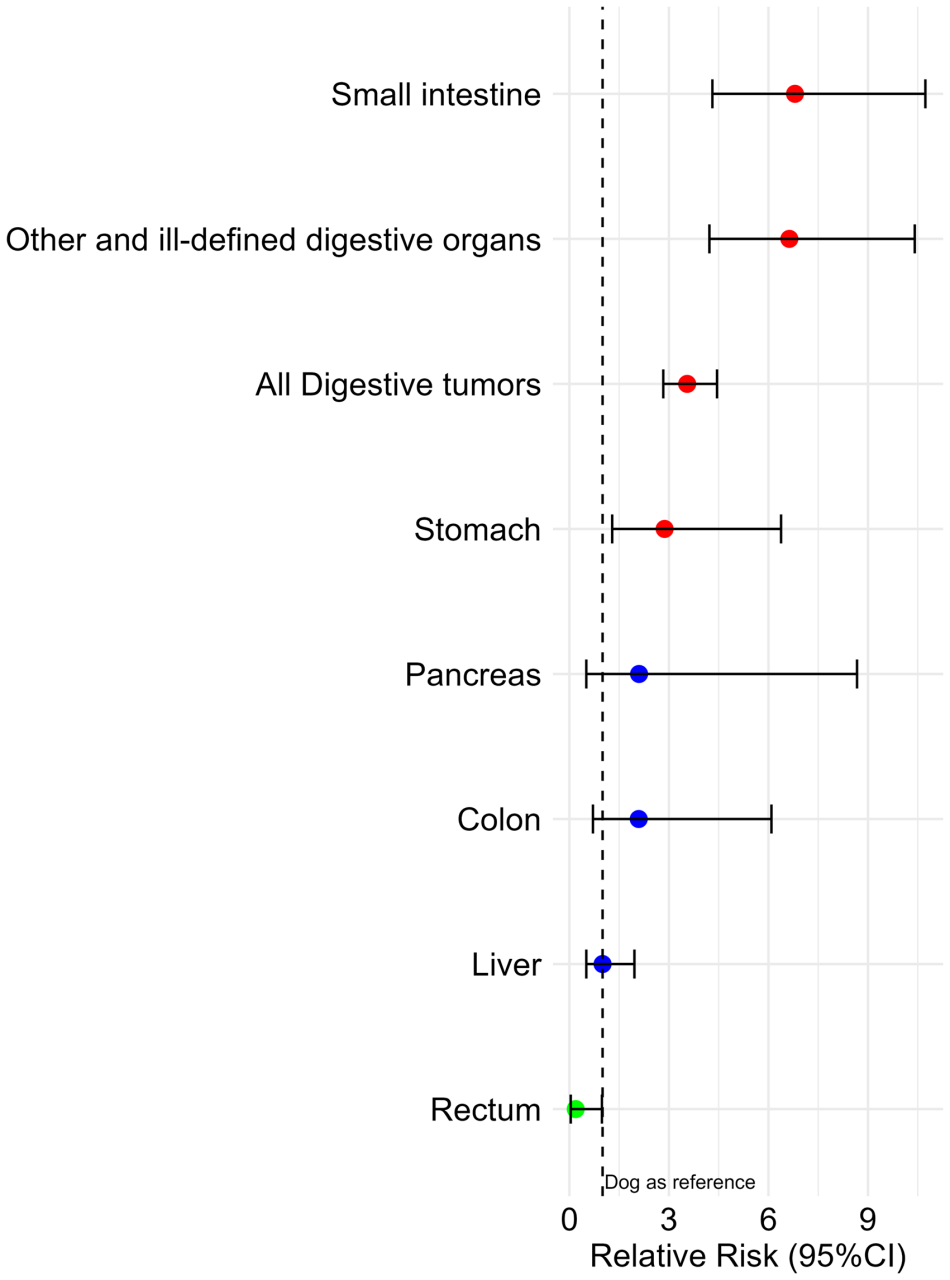
Relative risk and corresponding 95% confidence interval (95%CI) for digestive tumors in cats *versus* dogs.

Overall, cats have a significantly higher RR of developing digestive tract tumors (RR = 3.6 CI95% 2.83–4.45, *p* < 0.001), across multiple locations (Figure 3 and Supplementary Table S5), indicating that cats are over three times more likely to develop these neoplasms than dogs. Small intestine tumors show the highest species difference, with an RR of 6.8 (4.31–10.73, *p* < 0.001), followed by other and ill-defined digestive organ tumors (RR = 6.6, 4.22–10.41, *p* < 0.001). Gastric tumors are also more frequent in cats, with an RR of 2.9 (1.29–6.38, *p* < 0.001). In contrast, rectal tumors are more common in dogs, with an RR of 0.2 (0.04–0.98, *p* < 0.001), suggesting a fivefold lower risk in cats (Figure 3 and Supplementary Table S5).

**Figure 4:**
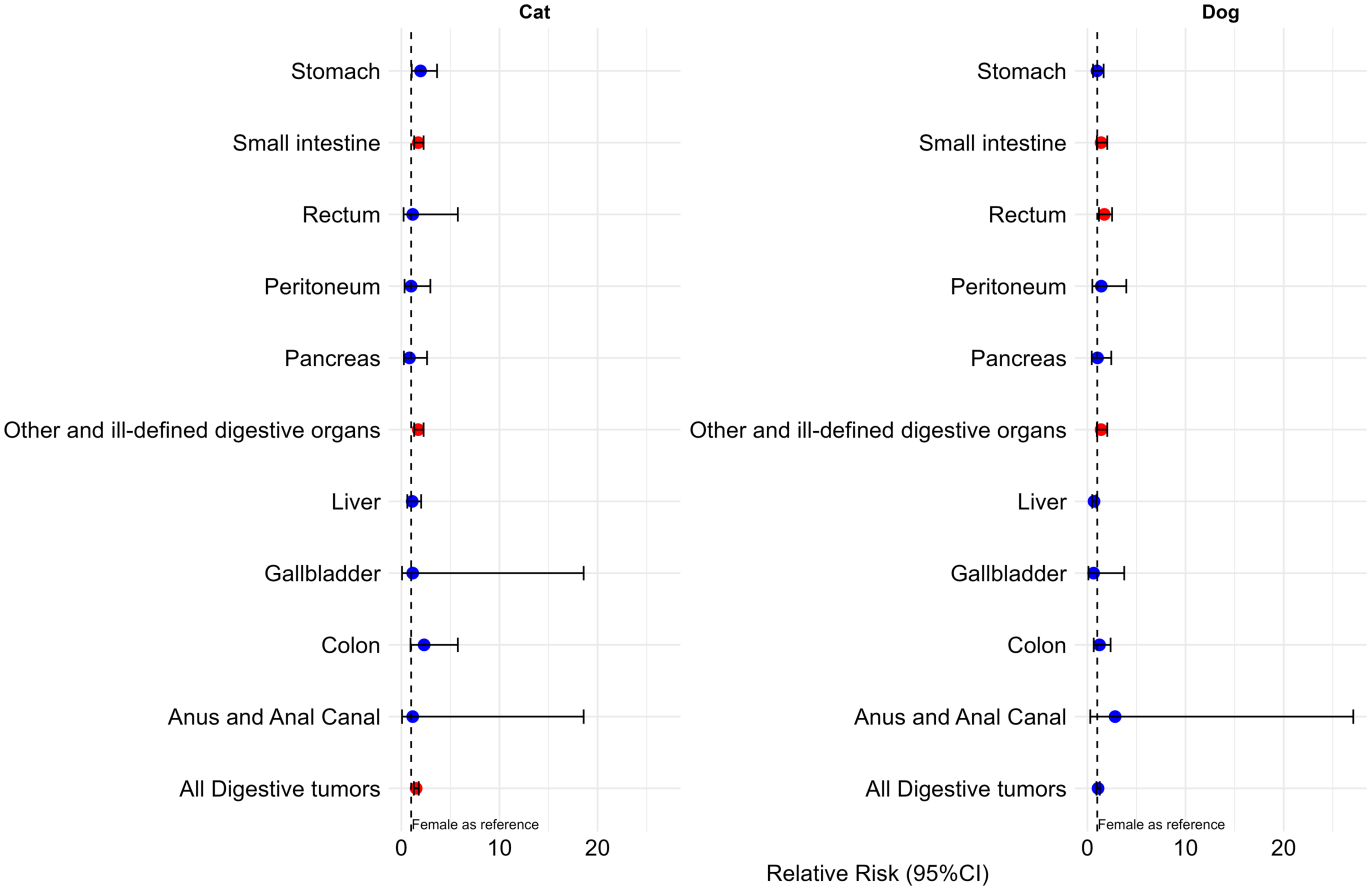
Relative risk and corresponding 95% confidence interval (95%CI) for digestive tumors in males *versus* female, in cats and dogs.

Figure 4 presents the analysis of sex as a risk of developing digestive tumors, showing that only male cats exhibit a higher risk of developing tumors in all digestive tract (RR = 1.5 CI95% 1.36–1.98, *p* < 0.001) (Supplementary Table S6). When analyzing tumor location, in both species, males present an increased risk for tumors in the small intestine (cats: 1.7 CI95% 1.44–2.05, *p* < 0.001; dogs: 1.4 CI95% 1.09–1.77, *p* < 0.001) and in other and ill-defined organs (cats: 1.7 CI95% 1.44–2.05, *p* < 0.001; dogs: 1.4 CI95% 1.09–1.77, *p* < 0.001). In dogs, males also show a higher risk specifically for tumors in the rectum (1.7 CI95% 1.37–2.16, *p* < 0.001) (Figure 4 and Supplementary Table S6).

**Figure 5:**
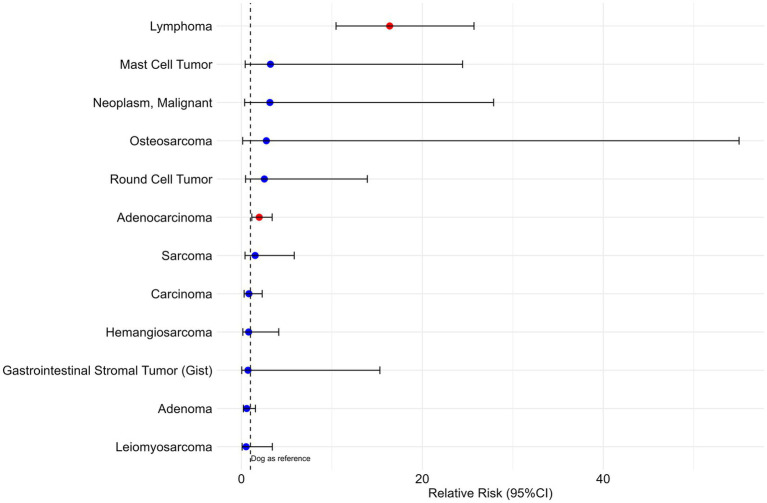
Relative risk and corresponding 95% confidence interval (95%CI) for different morphologies of digestive tumors in cats versus dogs.

Globally, and in comparison with dogs, cats presented a statistically significant 16× higher risk of developing GI lymphoma (RR = 16.4, 95%CI 10.46–25.71, *p*-value = 0.000) and a two times higher risk for adenocarcinoma (RR = 2.0, 95%CI 1.15–3.40, *p*-value = 0.014) (Figure 5 and Supplementary Table S7).

Additionally, male cats presented a higher lymphoma IR (IR = 19.0, 95% CI 16.63–21.37) than females (IR = 11.1, 95% CI 9.44–12.80) (Supplementary Table S8). Furthermore, male cats exhibited a 1.7-fold increased risk of developing lymphoma relative to females (RR = 1.7, 95%CI 1.40–2.08, *p* < 0.001) (Supplementary Table S9).

Among cats, carcinomas were significantly more prevalent in females compared to males (RR = 0.3, 95%CI 0.11–1.01, *p* < 0.001). No other statistically significant differences were observed between sexes in dogs (Supplementary Table S9).

**Figure 6:**
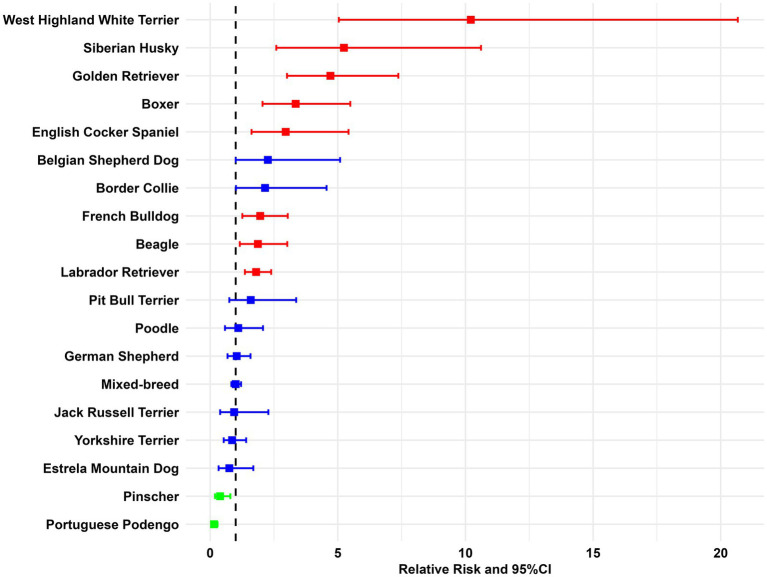
Canine breed-specific relative risk for digestive tract tumors (95% Confidence Intervals). Color red indicates breeds presenting significantly increased risk; blue denotes no significant differences compared to the reference group; and green, indicates breeds with significantly decreased risk.

The higher IR of digestive tract tumors was encountered in West Highland White Terrier (IR = 55 cases per 100.00), Siberian Husky (IR = 28.2 cases per 100.00), and Golden Retriever (IR = 25.3 cases per 100.00) canine breeds. On the other hand, a putative protective effect was noted in Pinschers (RR = 0.4, 0.19–0.79) and Portuguese Podengo (RR = 0.2, 0.08–0.29), when compared to mixed-breed dogs (Figure 6 and Supplementary Table S2).

**Figure 7:**
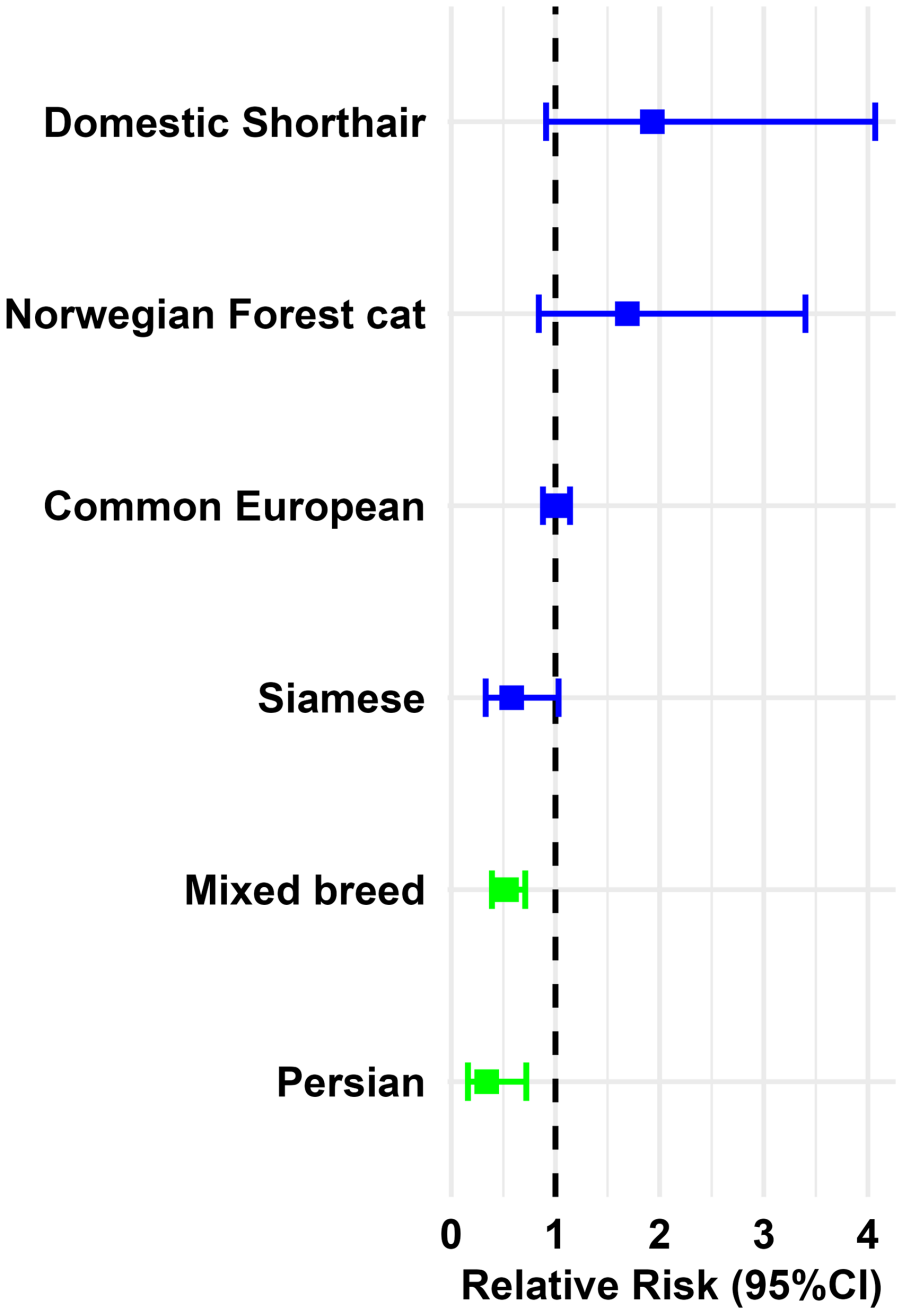
Feline breed-specific relative risk for digestive tract tumors (95% Confidence Intervals and *n* > 5). Color red indicates breeds presenting significantly increased risk; blue denotes no significant difference compared to the reference group; and green represents breeds with significantly decreased risk.

Domestic shorthair (IR = 43.8 cases per 100.00), Norwegian Forest (IR = 38.3 cases per 100.00), Common European (IR = 22.7 cases per 100.00) and Siamese (IR = 13.1 cases per 100.00) cats denoted no significant difference in IR of digestive tract tumors. On the other hand, mixed-breed (RR = 0.5, 0.39–0.71) and Persian cats (RR = 0.3, 0.16–0.72) exhibited a significantly decreased risk (Figure 7 and Supplementary Table S3) for developing digestive tract tumors.

### Spatial distribution

**Figure 8:**
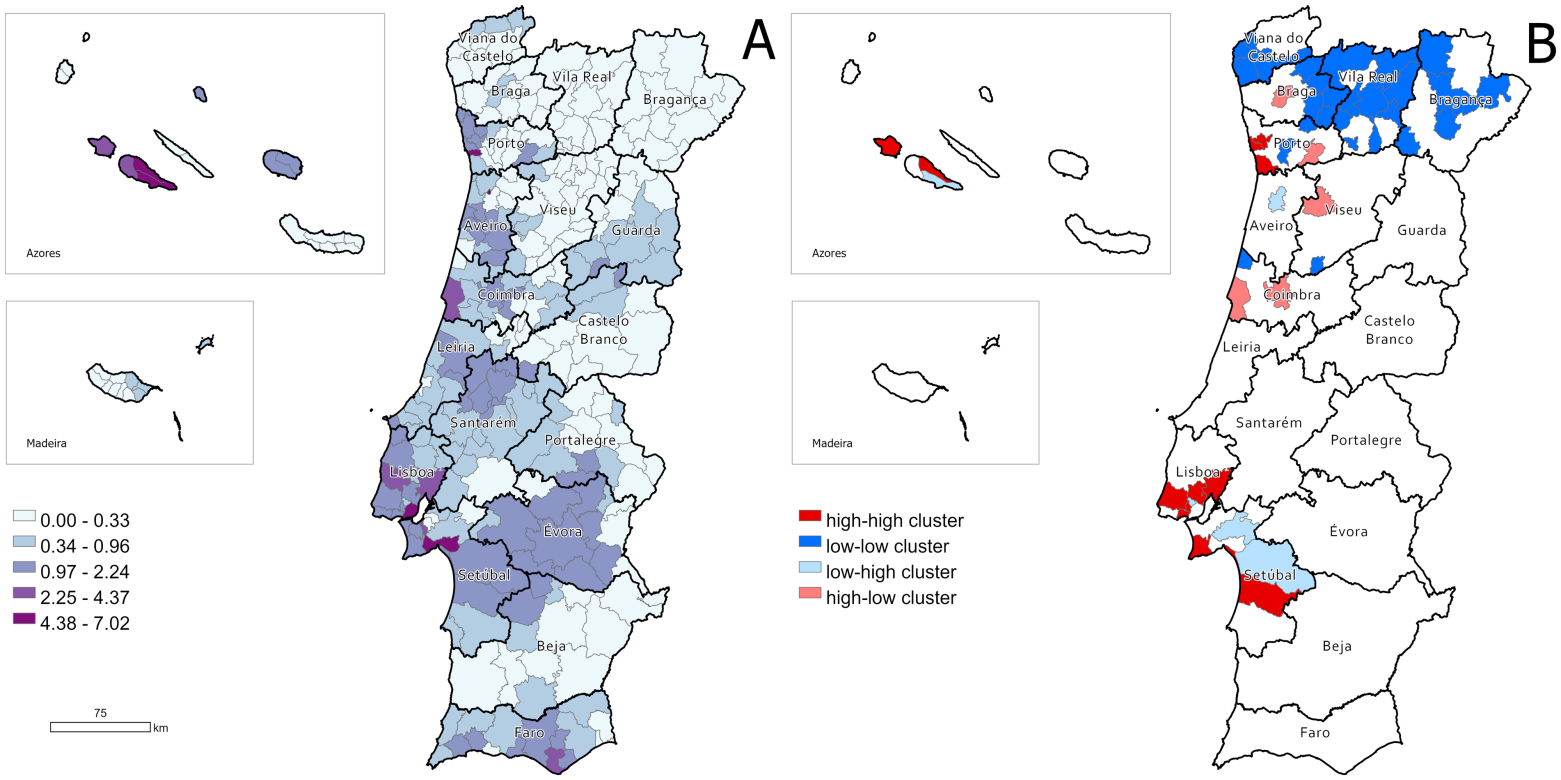
Geographical distribution of the standardized incidence ratios (SIR) of digestive tract tumors among dogs. **(A)** SIR according to municipality. **(B)** LISA cluster map highlighting geographical clusters and outliers.

The distribution of digestive tumors in dogs in Portugal, based on standardized incidence rates (SIR), is depicted in Figure 8. The pattern of the smoothed SIR exhibits a small yet statistically significant clustering (Moran’s *I* = 0.114, *p* = 0.01) (Figure 8A).

Figure 8B shows the results of LISA cluster map. The highest ratios were concentrated in the municipalities on the south and north coasts of the country, specifically in the highly urbanized metropolitan areas of Porto and Lisbon, where several high-high clusters were identified. Similar high-high clusters were also observed in the Azores islands. Considerable rates were also detected in the districts of Santarém and Setúbal. In contrast, lower ratios were mainly located in inner regions of the country, namely in more remote and inland municipalities located in the districts of Viana do Castelo, Braga, Vila Real, Viseu, and Bragança, which formed a large low-low cluster.

## Discussion

The analysis of a set of records relating to digestive tract tumors reported by various national laboratories is a valuable strategy because, in addition to allowing epidemiological comparisons between canine and feline species, it can reveal species-specific characteristics related with age and sex, topography, morphology, breed and tumor incidence. These findings will contribute to the understanding and enrichment of comparative oncology and may suggest the influence of certain genetic or behavioral factors, in addition to possible environmental causes (29, 30).

This study population included more male dogs and male cats than females, contrary to what was previously reported in an Italian study based on a cancer registry (30). Among both species and sexes, intact animals were more frequent, these being mainly dogs. This potential data gap in the reproductive status should be considered when assessing putative associations between reproductive status and the occurrence of digestive tract tumors.

Generally, cats tend to present digestive tract tumors later in life than dogs, which is in accordance with the literature (29, 30). In addition, as previously documented, cats are also more prone to developing intestinal tumors and are less affected with liver and intrahepatic bile ducts tumors than dogs (31–33).

Most dogs with digestive tumors were middle-aged to older dogs (34). The mean age of dogs with hepatic tumors was 10.8 years, which is in accordance with previous reports that mentioned an average age of 11 years for dogs diagnosed with hepatocellular neoplasia (32, 35–37). The mean age of dogs with rectal tumors was 8.3 years, which also is consistent with previous reports (38). For tumors located in the small intestine, herein the mean age of the dogs affected was 9.5 years, but some authors mentioned an average age of 11.9 years (5). The mean age of dogs with intestinal lymphoma, adenocarcinoma, and leiomyosarcoma were 7.5, 9.7 and 10.8 years, respectively. In contrast, another study documented mean ages of 8.5 years of dogs with intestinal adenocarcinoma and lymphoma, and 12 years for those with leiomyosarcoma (34).

Canine patients diagnosed with gastric tumors presented a mean age of 10.3 years. Specifically, dogs with gastric lymphoma had an average age of 11.6 years and those with adenocarcinoma had 10.3 years, and with leiomyoma 10.7 years. In comparison, another study reported a mean age of 7.3 years for dogs with gastric lymphoma, 9.5 years for those with adenocarcinoma, and 12.5 years for dogs diagnosed with gastric leiomyoma (34).

Overall, in the present study, the mean age of cats with digestive tumors was 10.5 years, and cats with tumors located in the small intestine and colon had a mean age of 10.6 years and 10.3 years, respectively. Rissetto et al., demonstrated that there is a gradual increase in the risk of developing intestinal neoplasia over the age of 7 years in cats (12). The development of malignant GI tumors increases with age in cats, with a mean age at diagnosis of 11.2 years, and those aged between 10 and 14 years (mean age of 10.8 years) having the highest risk of developing malignant intestinal tumors (38).

Interestingly, rectal tumors affected younger animals in both species, presenting the lowest mean age.

In dogs, the most affected digestive organs were, by descending order, liver and intrahepatic bile ducts, rectum, small intestine and stomach. In this species, and consistently with some of the available literature, the most frequent hepatic neoplasms diagnosed were carcinoma and adenoma (32, 35–37). Among carcinomas, hepatocellular carcinomas and cholangiocarcinoma were the most commonly identified types, whereas hepatocellular adenomas were the most frequent within adenomas. Supporting these findings, a UK study on canine hepatobiliary diseases found hepatocellular neoplasms to be the most common neoplastic liver condition (32). Another study reported hepatocellular carcinoma as the most frequent histological type in dogs (35). Similarly, a retrospective analysis from the University of Tokyo identified hepatocellular adenomas and hepatocellular carcinomas as the most prevalent canine proliferative hepatic diseases (37). Hemangiosarcoma ranked as the third most common hepatic tumor, while in other investigations it was the fourth primary nonepithelial tumor (37) or even the most common hepatic malignancy (39).

Rectum was predominantly affected by epithelial neoplasms, namely adenoma and adenocarcinoma which is consistent with the literature (5, 8, 34). In fact, 40% of intestinal adenocarcinomas are found in the colon and rectum (34). Both results are in accordance with a previous investigation (40), while other studies have shown adenocarcinoma as the predominant tumor (5, 38) and secondly adenoma (5). Nevertheless, Nucci et al. described carcinoma as the most common rectal masses in dogs (41). The descriptive analysis revealed the rectum as a relatively frequent anatomical site for tumor development in this species. Notably, adenomas represented most rectal tumors. This finding suggests that benign glandular proliferations are common in the canine rectum, which may reflect early clinical detection, a true epidemiological pattern, or both. Herein, most of the adenomas were classified as “adenomas, NOS,” followed by villous adenomas. Regarding adenocarcinomas, the majority were also classified as “adenocarcinomas, NOS” (not otherwise specified), which constrains the identification of their specific histological subtype.

Canine GI lymphomas correspond to 1.5–7.8% of all lymphoma forms (42, 43), being most often observed in small intestine than in stomach (44, 45), reflecting what is demonstrated in the present study. Herein, small intestine lymphomas ranked second, following adenocarcinoma. Other investigations demonstrated that small intestine is mostly affected by adenocarcinoma, adenoma and lymphoma (5, 34). Once again, most adenocarcinomas were classified as adenocarcinomas, NOS, making it impossible to determine their specific histological subtype. Leiomyosarcomas consisted of the third neoplasm in canine small intestine and have been also described as the second predominant neoplasm in the intestinal tract (8).

The stomach was mostly affected by adenocarcinoma, followed by leiomyoma being in line to what have been previously described (5, 8, 34, 46, 47). Most adenocarcinomas were also classified as “adenocarcinomas, NOS,” impairing again the histological subtype determination. Gastric lymphoma was the third neoplasm identified. Nevertheless, in a Czech Republic research it was recorded as the second most common and a breed predisposition in Dobermans was defended (34). In another study conducted by the Pathology Department of The Animal Medical Center, lymphoma was reported as the fourth most frequent canine gastric neoplasm (8). Carcinoma corresponded to the fourth most common gastric neoplasm. However, a German investigation on canine gastric neoplasia identified it as the most common tumor type, with adenocarcinoma being the second most frequently diagnosed (17). Nevertheless, that study did not specify the most common histological subtype, whereas in the present study, the predominant subtypes were carcinoma, NOS and signet ring cell carcinoma. In cats, and similarly to that previously described, carcinomas of the small intestine were common (12), followed by carcinomas located in the stomach, liver and intrahepatic bile ducts and colon.

Lymphomas were the most common tumor in cats, these results being consistent with previous studies (13, 48). On the other hand, Graf et al. demonstrated that adenocarcinoma was the main neoplasm and lymphoma was the second most common feline GI tumor in Swiss feline population (49) and another retrospective study based on incidence of feline tumors in the Japanese cat population indicated adenocarcinoma as the most frequent tumor in the cats’ alimentary system (50).

It is also well known that lymphoma is the most predominant intestinal tumor in cats (12, 51). Besides lymphoma, feline small intestine was also affected by adenocarcinoma (all adenocarcinoma NOS) and leiomyosarcoma. Other investigations assessing different cat populations from different countries (USA, Germany, Switzerland, and Italy) documented intestinal lymphoma as the most common neoplasm (12, 13, 49, 52) and adenocarcinoma as the second most common malignant tumor in cats’ intestine (12, 49, 52). In another Japanese study, leiomyosarcoma was the second most common malignant tumor in feline alimentary system (50). A German investigation reported small intestine as the most frequent site for leiomyosarcoma (13), being in line with our findings.

Lymphoma was also predominant in the feline stomach, resembling other studies (13, 52). Gastric adenocarcinomas and carcinomas were also found, being the stomach the least affected digestive tract site by these tumors, similar to what have been previously stated (13).

In cats, the liver and intrahepatic bile ducts were mainly affected by adenomas and lymphomas, which is consistent with findings from other studies assessing feline populations with hepatic tumors in various countries, including the UK, Japan, the Netherlands, Switzerland, Germany, and New Zealand (33, 37, 53, 54). Herein, only one case of hepatocellular carcinoma was found in cats (Supplementary Table S1), which is in accordance with Hirose et al. (37). However, some authors reported this tumor as the most common hepatic tumor (33, 50, 52, 54).

In colon, adenocarcinomas (mainly NOS) were predominant and lymphomas were the second most common neoplasm, which is in accordance with previous results (12). It is possible to confirm that, in cats, lymphomas were the most common in small intestine, followed by adenocarcinomas, while in colon the predominance is adenocarcinomas followed by lymphomas, being in line with what has been already reported (12, 13).

The high number of tumors in other and ill-defined digestive organs might be attributed to a lack of clinical data, the inability to identify the organ due to the small size of the biopsy, or due to the tumor’s advanced stage and destructive neoplastic characteristics, which can modify the tissue architecture.

Regarding breeds, these tumors occurred mainly in mixed-breed dogs, Labrador Retrievers, German Shepherds and French Bulldogs. These breeds are probably more likely predisposed to develop digestive tract neoplasms due to genetic factors. In fact, Grüntzig et al. described the incidence of neoplasms in the Swiss canine population and demonstrated that Shepherds, crossbreeds and Retrievers were the most frequent breeds affected (10). Taken together these results may suggest an overlapping risk profile linked to their known predisposition to other cancers.

The observed differences in mean age across dog breeds may reflect a breed-specific survival patterns in this population, aligning with previous research on canine life expectancy at birth in Portugal, which reported English Cocker Spaniel and Yorkshire Terrier to have the highest lifespans, exceeding 11 years; and French Bulldog with the lowest life expectancy, under 7 years (55). It is known that age and breed remain risk factors for tumor development in dogs (56). These findings could suggest that age plays a modulating role in breed-related tumor risk, since some breeds may develop digestive tract tumors earlier than others probably due to accelerated aging processes or other breed-specific factors. Nevertheless, several investigations reported a higher incidence of gastric tumors in Rough Collie, Staffordshire Bull Terrier, Belgian Shepherd, Belgian Tervuren, Bouvier des Flandres, Groenendael, Collie, Chow–Chow, Poodle, Norwegian Elkhound (17, 46, 47, 57–60). While Collie and German Shepherd have been associated with intestinal tumors (8). Moreover, another study evidenced a breed predisposition for gastric tumors in the Doberman, Belgian Shepherd and Leonberger and reported another breed predisposition for intestinal tumors in Pug, Leonberger, English Setter, Hovawart, Doberman (34). In Japan, Jack Russell Terriers and Miniature Dachshunds were identified as the breeds most frequently affected by gastric and intestinal tumors, respectively (5), and Golden Retrievers and Shiba were most commonly associated with hepatocellular adenoma. Shih Tzus were predominantly affected by hepatocellular carcinoma, followed by Yorkshire Terriers (37).

In the present study, there is an overrepresentation of the Portuguese Podengo breed, likely due to the fact that a significant number of animals are registered as Portuguese Podengo in the SIAC, despite not all of them truly belonging to this breed. This discrepancy may stem from misclassification or inaccuracies in breed identification during the registration process, which could impact the reliability of breed-specific data in the study.

A feline breed predisposition for digestive tract tumors was not observed in this study, since about 72.5% of the cats were generically classified as “Common European” breed. These findings are in agreement with a previous investigation from Germany, which retrospectively analyzed the frequency, histomorphology, and immunohistochemical characteristics of feline gastrointestinal neoplasms submitted for routine diagnosis, and reported that 80% of the cases involved domestic short-haired cats or mixed breeds (13). Although some studies reported an overrepresentation of Siamese cats (12, 49, 61, 62). Siamese cats were uncommon in this study, being in line with previous research (13).

Our findings underscore notable differences in the incidence and relative risks of digestive tract neoplasms between dogs and cats, as well as between sexes within each species. The incidence of these tumors was considerably higher in cats than in dogs, suggesting a species-specific predisposition. A previous report stated that cats have a higher risk of malignant tumors compared to dogs (63), being most of the tumors in GI tract malignant (49, 64).

In both species, males demonstrate a higher RR to develop these tumors (in particular small intestine neoplasms) compared to females, which is in accordance with another study that demonstrated that males have a higher proportion of intestinal tract neoplasms (29). Yet, it has also been reported no significant difference in the odds between male and female cats (49). However, some studies suggest that sex do not play a major role in the frequency of tumor occurrence (34, 37). On the other hand, another research revealed that males have a higher proportion of intestinal tract tumors compared to females (29). Saito et al. indicated that large intestinal neoplasms are more frequent in male dogs, while gastric and small intestine tumors do not exhibit a sex preference (5). Patnaik et al. showed that gastric tumors, hepatocellular carcinoma and sarcoma occurred more often in male dogs (8, 35).

In the present study, tumors of the small intestine were not exclusively associated with cats, suggesting that this finding is not species-specific but rather related to sex, as both species showed a higher prevalence of cases in males. This observation raises important questions regarding potential underlying factors. One possibility is hormonal influence, as males may be predisposed to certain tumor types due to the effects of sex-related hormones. Additionally, the lower rates of castration could play a role, as intact males may experience prolonged exposure to hormonal fluctuations that could promote tumor development.

Studies in humans have shown that tumors of the small intestine are also more commonly observed in males than in females (65–67). Furthermore, in humans, colorectal cancer (CRC) demonstrates a higher incidence in males compared to females, where sexual dimorphism in CRC rates and survival is well-documented, suggesting a protective role of the sex steroid hormone estrogen in CRC development (68, 69). These observations may provide insight into the higher risk of intestinal tumors observed in male dogs and cats in present study, raising the possibility that hormonal differences between sexes, including the absence of estrogen’s protective effects, could contribute to the increased prevalence in males. Further research is required to explore these hypothesis and associations and clarify their underlying mechanisms.

This pattern in male animals was not observed in other digestive organs may be due to a lack of sufficient data for those other sites, limiting the ability to draw definitive conclusions about sex-based differences across the entire digestive system.

Cats exhibited a higher RR to develop all digestive tract tumors, small intestine and gastric tumors, when compared to dogs. This significant difference reinforces the idea of a species-specific predilection, possibly linked physiological or environmental factors unique to cats. Chronic inflammation plays a key role as a risk factor in the development of GI malignancies (70). Feline chronic enteropathies, encompassing conditions like inflammatory bowel disease (IBD), could play a major role in cats, given its known association with GI tumor development (71). Additionally, the comparative analysis across species showed that cats had a higher risk of lymphoma compared to dogs, which has also been previous reported (29), highlighting once again the species-specific nature of tumor development. Regarding morphology the results also suggest a potential sex-specific biological mechanisms influencing tumor development in felines, possibly linked to hormonal status, immune function, or environmental exposures. Male cats exhibited a significantly higher risk of developing lymphoma, whereas carcinomas were more prevalent in females. Interestingly, no statistically significant differences in tumor incidence were observed between male and female dogs.

Additionally, our study highlights important breed-specific differences in the IR and RR of developing digestive tract tumors in both dogs and cats. Among dogs, West Highland White Terriers, Siberian Huskies and Golden Retrievers exhibited the highest risk, suggesting a potential genetic susceptibility that warrants early screening and preventive measures. In contrast, Pinschers and Portuguese Podengos demonstrated a protective effect, indicating possible genetic or physiological factors that may reduce tumor susceptibility. Similarly, in cats, domestic shorthairs, Norwegian Forest cats, common European and Siamese showed no significant differences in incidence rates, while mixed-breed and Persian exhibited a significant lower risk. Understanding these breed-specific variations is crucial for improving early detection strategies.

There is limited information on the geographical distribution of digestive tract tumors in the human Portuguese population. However, colorectal cancer incidence is higher in the North and Center regions, with clusters along the West Coast, including Lisbon and Vale do Tejo. Other areas show only small, isolated high-risk zones surrounded by lower-risk regions (72). Similarly, our spatial analysis of digestive tract tumor incidence in dogs across Portugal reveals significant geographic clustering, as evidenced by the smoothed SIR and LISA clusters. The presence of high-high clusters in densely populated and urbanized regions, such as the metropolitan areas of Porto and Lisbon, suggests a potential influence of environmental factors, lifestyle exposures, or differences in veterinary healthcare access. Additionally, higher IR observed in certain inland districts like Évora and Santarém reinforce the need to investigate potential regional risk factors. Conversely, the large low-low cluster observed in the northern and inland regions, particularly in the districts of Viana do Castelo, Braga, Vila Real, and Bragança, indicates a significantly lower incidence in these areas, which may reflect differences in demographic, genetic, or environmental factors affecting canine populations. The statistically significant spatial autocorrelation confirms that the distribution of these tumors is not random, highlighting the importance of further epidemiological research to assess environmental, genetic, and veterinary care-related influences on tumor prevalence across different regions.

## Limitations

A significant limitation of this study is the underreporting within Vet-OncoNet and the SIAC system. In SIAC databases’ this encompasses the underreporting of cats and the deceased animals, which tend to be underrepresented in the data. Furthermore, cases lacking a definitive diagnosis and data from laboratories not affiliated with the network are likely excluded. Despite these limitations, the study provides the most accurate reflection of reality currently available, although it can be further improved.

Not all laboratories perform IHC analysis, which may lead to potential misclassification of some cases. This limitation should be considered especially when interpreting the results related to leiomyosarcoma incidence.

Regarding tumor morphology, the majority were classified as “not otherwise specified” (NOS), which significantly limited the interpretation of our results concerning their histological subtypes. This may be attributed to the lack of detailed pathological characterization. Future studies should incorporate more refined histopathological techniques to enhance subtype classification in order to produce a comprehensive descriptive analysis of these pathologies which is essential for advancing comparative pathology and understanding species-specific and cross-species patterns of tumor development.

Another limitation encompasses the reproductive status (spayed or intact) of the animals, which must be interpreted with caution due to insufficient information in some cases. Nonetheless, it is crucial to assess this data in future research, since it can provide valuable insights into the potential relationship between the reproductive factors and the development of digestive tract tumors.

However, it is important to note that the low cluster observed in Vila Real and its surrounding areas may be influenced and partially explained by the lack of available data from these locations, which could lead to an underestimation of the true incidence rates. Furthermore, the possibility of greater access to diagnostics in high-incidence areas cannot be ruled out as a contributing factor. Despite these limitations, the observed patterns provide valuable insights and contribute to growing evidence supporting the role of environmental factors in the development of digestive tract tumors in dogs, warranting further investigation into potential regional risk factors.

## Conclusion

This study reveals significant differences in digestive tract tumors between dogs and cats, related to age, sex, breed, tumor location and morphology, suggesting distinct profiles for each species. This variation in tumor incidence proposes a genetic background, as well as environmental factors, being important the implementation of species-specific strategies for diagnosing and preventing such neoplasms.

Additionally, the spatial patterns in both humans and canine populations suggest the potential role of shared environmental, demographic, or healthcare-related factors influencing digestive tract tumor incidence in Portugal.

The results herein presented constitute the first epidemiological study focusing on digestive tract tumors in the Portuguese dog and cat population. Given the limited data available for this specific geographical area, these results may contribute to the characterization of neoplastic entities and their distribution in Portuguese canine and feline populations, enhancing our understanding of the potential risks and outcomes. Studying the epidemiology and distribution patterns of malignancies in dogs and cats is essential not only for improving animal health, but also for informing public health strategies.

## Data Availability

The datasets presented in this article are not readily available because the data are the property of the Vet-OncoNet network and are accessible upon reasonable request, subject to authorization by the network. Requests to access the datasets should be directed to kcpinello@icbas.up.pt.
