# Supplementary material for: Comparative epidemiological analysis of tumors of the digestive system in dogs and cats
Source: Front Vet Sci. 2025 Nov 13;12:1701594. doi: 10.3389/fvets.2025.1701594 (PMC12661348; doi:10.3389/fvets.2025.1701594)
Supplement: Supplementary file 1 [file Table_1.DOCX]

Supplementary Material

Table S1: Morphological classification of digestive tract tumors in dogs and cats.

|  | **Total** | **Dogs** | **Cats** |
| --- | --- | --- | --- |
|  | **n (%)** | **n (%)** | **n (%)** |
| **TOTAL** | 1075 (100) | 576 (100) | 499 (100) |
| **Lymphomas** | 512 (42.2) | 94 (15.2) | 418 (70.1) |
| Malignant lymphoma (non-Hodgkin), NOS _(9591/3)_ | 334 (27.5) | 60 (9.7) | 274 (46.0) |
| Intestinal T‐cell lymphoma (enteropathy associated) _(9717/3)_ | 75 (6.2) | 9 (1.5) | 66 (11.1) |
| Peripheral T-cell lymphoma, NOS _(9702/3)_ | 41 (3.4) | 6 (1.0) | 35 (5.9) |
| B-cell lymphoblastic leukemia/lymphoma _(9811/3)_ | 18 (1.5) | 7 (1.1) | 11 (1.8) |
| B-cell lymphoma, NOS _(9591.1/3)_ | 11 (0.9) | 4 (0.6) | 7 (1.2) |
| B-cell chronic lymphocytic leukemia/small (cell) lymphocytic lymphoma _(9823/3)_ | 9 (0.7) | 0 | 9 (1.5) |
| Hepatosplenic T-cell lymphoma _(9716/3)_ | 7 (0.6) | 3 (0.5) | 4 (0.7) |
| T-cell Large granular lymphocytic (LGL) leukemia/lymphoma _(9831/3)_ | 7 (0.6) | 1 (0.2) | 6 (1.0) |
| Diffuse large B‐cell lymphoma (DLBCL) _(9680/3)_ | 4 (0.3) | 2 (0.3) | 2 (0.3) |
| T‐cell lymphoma, NOS _(9591.2/3)_ | 4 (0.3) | 1 (0.2) | 3 (0.5) |
| T‐cell‐rich large B‐cell lymphoma _(9688/3)_ | 2 (0.2) | 1 (0.2) | 1 (0.2) |
| **Adenocarcinomas** | 230 (19.0) | 149 (24.1) | 81 (13.6) |
| Adenocarcinoma, NOS _(8140/3)_ | 164 (13.5) | 103 (16.7) | 61 (10.2) |
| Mucinous adenocarcinoma _(8480/3)_ | 28 (2.3) | 19 (3.1) | 9 (1.5) |
| Tubular carcinoma _(8211/3)_ | 15 (1.2) | 9 (1.5) | 6 (1.0) |
| Tubulopapillary carcinoma _(8263/3)_ | 10 (0.8) | 8 (1.3) | 2 (0.3) |
| Trabecular adenocarcinoma _(8190/3)_ | 4 (0.3) | 4 (0.6) | 0 |
| Acinar adenocarcinoma _(8550/3)_ | 3 (0.2) | 1 (0.2) | 2 (0.3) |
| Papillary adenocarcinoma _(8260/3)_ | 2 (0.2) | 2 (0.3) | 0 |
| Tubular adenocarcinoma _(8811/3)_ | 2 (0.2) | 1 (0.2) | 1 (0.2) |
| Adenocarcinoma in situ, NOS _(8140/3)_ | 1 (0.1) | 1 (0.2) | 0 |
| Cystic adenocarcinoma _(8440/3)_ | 1 (0.1) | 1 (0.2) | 0 |
| **Adenomas** | 140 (11.5) | 120 (19.4) | 20 (3.4) |
| Adenoma, NOS _(8140/0)_ | 46 (3.8) | 43 (7.0) | 3 (0.5) |
| Hepatocellular adenoma _(8170/0)_ | 46 (3.8) | 41 (6.6) | 5 (0.8) |
| Villous adenoma, NOS _(8261/0)_ | 22 (1.8) | 19 (3.1) | 3 (0.5) |
| Tubular adenoma _(8211/0)_ | 8 (0.7) | 8 (1.3) | 0 |
| Papillary adenoma _(8260/0)_ | 7 (0.6) | 6 (1.0) | 1 (0.2) |
| Cholangiocellular adenoma _(8160/0)_ | 5 (0.4) | 1 (0.2) | 4 (0.7) |
| Bile duct cystadenoma _(8161/0)_ | 4 (0.3) | 0 | 4 (0.7) |
| Tubulopapillary adenoma _(8263/0)_ | 2 (0.2) | 2 (0.3) | 0 |
| **Carcinomas** | 99 (8.2) | 81 (13.1) | 18 (3.0) |
| Hepatocellular carcinoma, NOS _(8170/3)_ | 35 (2.9) | 33 (5.3) | 2 (0.3) |
| Carcinoma, NOS _(8010/3)_ | 19 (1.6) | 12 (1.9) | 7 (1.2) |
| Cholangiocarcinoma _(8160/3)_ | 14 (1.2) | 11 (1.8) | 3 (0.5) |
| Neuroendocrine carcinoma, NOS _(8246/3)_ | 10 (0.8) | 8 (1.3) | 2 (0.3) |
| Carcinoma, anaplastic, NOS _(8021/3)_ | 4 (0.3) | 4 (0.6) | 0 |
| Hepatocellular carcinoma, clear cell type _(8174/3)_ | 3 (0.2) | 3 (0.5) | 0 |
| Islet cell carcinoma, NOS _(8150/3)_ | 3 (0.2) | 3 (0.5) | 0 |
| Signet ring cell carcinoma _(8490/3)_ | 3 (0.2) | 3 (0.5) | 0 |
| Carcinoma in situ, NOS _(8010/2)_ | 2 (0.2) | 2 (0.3) | 0 |
| Carcinoma, undifferentiated, NOS _(8020/3)_ | 2 (0.2) | 1 (0.2) | 1 (0.2) |
| Solid adenocarcinoma _(8230/3)_ | 2 (0.2) | 1 (0.2) | 1 (0.2) |
| Solid carcinoma _(8230/3)_ | 1 (0.1) | 0 | 1 (0.2) |
| Squamous cell carcinoma, NOS _(8070/3)_ | 1 (0.1) | 0 | 1 (0.2) |
| **Sarcomas** | 35 (3.3) | 26 (4.5) | 9 (1.8) |
| Sarcoma, NOS _(8800/3)_ | 16 (1.3) | 10 (1.6) | 6 (1.0) |
| Soft tissue tumor, NOS _(8800.0/1)_ | 12 (1.0) | 11 (1.8) | 1 (0.2) |
| Undifferentiated sarcoma _(8805/3)_ | 9 (0.7) | 5 (0.8) | 4 (0.7) |
| Histiocytic sarcoma _(9755/3)_ | 4 (0.3) | 3 (0.5) | 1 (0.2) |
| Leiomyosarcoma, NOS _(8890/3)_ | 41 (3.4) | 36 (5.8) | 5 (0.8) |
| Hemangiosarcoma, NOS _(9120/3)_ | 39 (3.2) | 32 (5.2) | 7 (1.2) |
| Round cell tumor, NOS _(8006.1/1)_ | 22 (1.8) | 13 (2.1) | 9 (1.5) |
| Leiomyoma, NOS _(8890/0)_ | 18 (1.5) | 18 (2.9) | 0 |
| Mast cell tumor, NOS _(9740/1)_ | 15 (1.2) | 8 (1.3) | 7 (1.2) |
| Neoplasm, malignant _(8000/3)_ | 13 (1.1) | 7 (1.1) | 6 (1.0) |
| Gastrointestinal stromal tumor (GIST) _(8936/3)_ | 12 (1.0) | 10 (1.6) | 2 (0.3) |
| **Insulinomas** | 7 (0.6) | 5 (0.8) | 2 (0.3) |
| Insulinoma, malignant _(8151/3)_ | 5 (0.4) | 3 (0.5) | 2 (0.3) |
| Insulinoma, NOS _(8151/0)_ | 2 (0.2) | 2 (0.3) | 0 |
| **Osteosarcomas** | 7 (0.6) | 4 (0.6) | 3 (0.5) |
| Extraskeletal osteosarsoma _(9180/3)_ | 2 (0.2) | 1 (0.2) | 1 (0.2) |
| Osteosarcoma, NOS _(9180/3)_ | 5 (0.4) | 3 (0.5) | 2 (0.3) |
| Hemangioma, NOS _(9120/0)_ | 4 (0.3) | 2 (0.3) | 2 (0.3) |
| Mesothelioma, benign _(9050/0)_ | 3 (0.2) | 0 | 3 (0.5) |
| Plasmacytoma, extramedullary (digestive tract) _(9734/1)_ | 3 (0.2) | 3 (0.5) | 0 |
| Liposarcoma, NOS _(8850/3)_ | 2 (0.2) | 2 (0.3) | 0 |
| Myxosarcoma, NOS _(8840/3)_ | 2 (0.2) | 2 (0.3) | 0 |
| Plasma cell myeloma _(9732/3)_ | 1 (0.1) | 1 (0.2) | 0 |
| Papilloma, NOS _(8050/0)_ | 1 (0.1) | 1 (0.2) | 0 |

Table S2: Incidence and relative risk of digestive tract tumors across dog breeds.

|  | **Total** | | **IR*100,00 dogs (95% CI)** | **RR (95% CI)** | ***p*-value** |
| --- | --- | --- | --- | --- | --- |
| **Breed** | **n (%)** | **Mean age (SD)** |  |  |  |
| Mixed breed | 220 (35.7) | 10.6 (3.0) | 5.4 | ref | - |
| Labrador Retriever | 61 (9.9) | 9.5 (2.8) | 9.7 | 1.8 (1.36-2.39) | <0.001 |
| German Shepherd | 24 (3.9) | 8.0 (1.9) | 5.6 | 1.0 (0.68-1.58) | 0.940 |
| French Bulldog | 22 (3.6) | 7.0 (2.6) | 10.5 | 2.0 (1.26-3.04) | <0.001 |
| Golden Retriever | 21 (3.4) | 10.5 (1.9) | 25.3 | 4.7 (3.01-7.37) | <0.001 |
| Beagle | 18 (2.9) | 7.6 (2.5) | 10.1 | 1.9 (1.16-3.02) | <0.001 |
| Boxer | 17 (2.8) | 8.2 (3.0) | 18.0 | 3.4 (2.05-5.49) | <0.001 |
| Yorkshire Terrier | 17 (2.8) | 10.3 (2.7) | 4.6 | 0.9 (0.53-1.41) | 0.619 |
| English Cocker Spaniel | 11 (1.8) | 11.6 (1.1) | 16.0 | 3.0 (1.62-5.42) | <0.001 |
| Poodle | 10 (1.6) | 9.8 (2.2) | 5.9 | 1.1 (0.58-2.07) | 0.892 |
| Portuguese Podengo | 9 (1.5) | 9.3 (3.4) | 0.8 | 0.2 (0.08-0.29) | <0.001 |
| Miniature Pinscher | 8 (1.3) | 9.6 (2.1) | 2.1 | 0.4 (0.19-0.79) | <0.001 |
| Siberian Husky | 8 (1.3) | 12.1 (1.9) | 28.2 | 5.2 (2.59-10.61) | <0.001 |
| West Highland White Terrier | 8 (1.3) | 11.4 (1.3) | 55.0 | 10.2 (5.04-20.67) | <0.001 |
| Pit Bull Terrier | 7 (1.1) | 9.5 (3.8) | 8.6 | 1.6 (0.75-3.37) | 0.323 |
| Border Collie | 7 (1.1) | 8.9 (3.7) | 11.6 | 2.2 (1.01-4.56) | 0.077 |
| Estrela Mountain Dog | 6 (1.0) | 8.0 (3.9) | 4.0 | 0.8 (0.33-1.69) | 0.599 |
| Belgian Shepherd | 6 (1.0) | 8.7 (1.5) | 12.2 | 2.3 (1.00-5.09) | 0.084 |
| Jack Russel Terrier | 5 (0.8) | 9.2 (2.7) | 5.1 | 0.9 (0.39-2.28) | 1.000 |
| Portuguese Pointer | 4 (0.6) | 10.8 (2.8) | 7.5 | 1.4 (0.52-3.74) | 0.719 |
| Bull Terrier | 4 (0.6) | 10.7 (1.2) | 15.0 | 2.8 (1.04-7.50) | 0.088 |
| Akita | 4 (0.6) | 7.8 (2.8) | 87.8 | 16.3 (6.07-43.87) | <0.001 |
| Pekingese | 4 (0.6) | 8.5 (3.3) | 2.1 | 0.4 (0.15-1.08) | 0.084 |
| Shar-pei | 3 (0.5) | 8.3 (4.0) | 10.1 | 1.9 (0.60-5.87) | 0.478 |
| English Bulldog | 3 (0.5) | 8.7 (2.3) | 5.7 | 1.1 (0.34-3.31) | 1.000 |
| Shih Tzu | 3 (0.5) | 12.3 (2.1) | 4.4 | 0.8 (0.26-2.56) | 0.939 |
| Cane Corso | 3 (0.5) | 7.7 (4.2) | 9.8 | 1.8 (0.58-5.69) | 0.512 |
| Chihuahua | 3 (0.5) | 9.0 (1.7) | 1.8 | 0.3 (0.11-1.06) | 0.077 |
| Basset Hound | 3 (0.5) | 9.0 (1.7) | 20.4 | 3.8 (1.22-11.87) | 0.056 |
| Rhodesian Ridgeback | 3 (0.5) | 10.3 (0.6) | 18.8 | 3.5 (1.12-10.90) | 0.079 |
| Teckel | 2 (0.3) | 10.0 (0.0) | 2.4 | 0.5 (0.11-1.81) | 0.356 |
| Pug | 2 (0.3) | 8.5 (3.5) | 5.9 | 1.1 (0.27-4.39) | 1.000 |
| Bernese Mountain Dog | 2 (0.3) | 8.0 | 25.2 | 4.7 (1.16-18.83) | 0.103 |
| Standard Schnauzer | 2 (0.3) | 9.5 (2.1) | 14.2 | 2.6 (0.65-10.58) | 0.399 |
| Whippet | 2 (0.3) | 9.5 (0.7) | 35.9 | 6.7 (1.66-26.84) | <0.001 |
| Great Dane | 2 (0.3) | 2.0 (1.4) | 7.4 | 1.4 (0.34-5.51) | 0.977 |
| Brittany Spaniel | 2 (0.3) | 9.5 (2.1) | 1.1 | 0.2 (0.05-0.80) | <0.001 |
| Alentejo Mastiff | 2 (0.3) | 3.5 (2.1) | 1.2 | 0.2 (0.05-0.89) | <0.001 |
| Cavalier King Charles Spaniel | 1 (0.2) | 10.0 | 7.1 | 1.3 (0.19-9.41) | 1.000 |
| American Staffordshire Terrier | 1 (0.2) | 9.0 | 3.9 | 0.7 (0.10-5.21) | 1.000 |
| Pointer | 1 (0.2) | 7.0 | 2.1 | 0.4 (0.05-2.78) | 0.503 |
| Bullmastiff | 1 (0.2) | 6.0 | 27.3 | 5.1 (0.71-36.23) | 0.496 |
| Japanese Spitz | 1 (0.2) | 11.0 | 78.6 | 14.6 (2.05-104.18) | 0.100 |
| Bordeaux Mastiff | 1 (0.2) | 7.0 | 13.0 | 2.4 (0.34-17.26) | 0.893 |
| Pyrenean Mountain Dog | 1 (0.2) | 13.0 | 75.5 | 14.0 (1.97-100.12) | 0.109 |
| Rottweiler | 1 (0.2) | 9.0 | 1.7 | 0.3 (0.04-2.21) | 0.332 |
| Tibetan Mastiff | 1 (0.2) | 10.0 | 38.6 | 7.2 (1.01-51.13) | 0.336 |
| Rough Collie | 1 (0.2) | 0.0 | 10.9 | 2.0 (0.28-14.40) | 0.994 |
| German Spitz | 1 (0.2) | 10.0 | 36.9 | 6.9 (0.96-48.85) | 0.355 |
| Doberman | 1 (0.2) | 9.0 | 4.5 | 0.8 (0.12-5.99) | 1.000 |
| Springer Spaniel | 1 (0.2) | 12.0 | 23.4 | 4.4 (0.61-31.02) | 0.574 |
| Miniature Schnauzer | 1 (0.2) | 13.0 | 7.1 | 1.3 (0.19-9.41) | 1.000 |
| Portuguese Water Dog | 1 (0.2) | 12.0 | 5.0 | 0.9 (0.13-6.63) | 1.000 |
| English Setter | 1 (0.2) | 7.0 | 2.2 | 0.4 (0.06-2.85) | 0.530 |
| Foz Terrier | 1 (0.2) | 8.0 | 8.9 | 1.7 (0.23-11.84) | 1.000 |
| Cairn Terrier | 1 (0.2) | 10.0 | 206.6 | 38.4 (5.38-273.83) | <0.001 |
| Dalmatian | 1 (0.2) | 3.0 | 3.8 | 0.7 (0.10-4.99) | 1.000 |

Table S3: Incidence and relative risk of digestive tract tumors across cat breeds

|  | **Total** | | **IR*100,00 cats (95% CI)** | **RR (95% CI)** | ***p*-value** |
| --- | --- | --- | --- | --- | --- |
| **Breed** | **n (%)** | **Mean age (SD)** |  |  |  |
| Common European | 432 (72.5) | 10.5 (3.3) | 22.7 | 1.0 (0.88-1.14) | 1.000 |
| Mixed breed | 49 (8.2) | 10.3 (3.5) | 12.0 | 0.5 (0.39-0.71) | <0.001 |
| Siamese | 12 (2.0) | 11.8 (1.7) | 13.1 | 0.6 (0.33-1.03) | 0.075 |
| Norwegian Forest cat | 8 (1.3) | 8.0 (3.3) | 38.3 | 1.7 (0.84-3.40) | 0.210 |
| Domestic Shorthair | 7 (1.2) | 11 (1.5) | 43.8 | 1.9 (0.91-4.07) | 0.134 |
| Persian | 7 (1.2) | 10.7 (2.3) | 7.6 | 0.3 (0.16-0.72) | <0.001 |
| Bengal | 3 (0.5) | 12.0 (0.0) | 41.4 | 1.8 (0.59-5.70) | 0.506 |
| Maine Coon | 2 (0.3) | 6.0 (2.8) | 12.5 | 0.6 (0.14-2.21) | 0.554 |
| British Shorthair | 2 (0.3) | 6.5 (3.5) | 3.6 | 0.2 (0.04-0.64) | <0.001 |
| Birman | 1 (0.2) | 8.0 | 63.9 | 2.8 (0.40-20.07) | 0.808 |
| Chartreux | 1 (0.2) | 7.0 | 89.0 | 3.9 (0.55-27.89) | 0.628 |
| Ragdoll | 1 (0.2) | 11.0 | 20.6 | 0.9 (0.13-6.48) | 1.000 |
| Sphynx | 1 (0.2) | 2.0 | 7.0 | 0.3 (0.04-2.21) | 0.336 |
| Turkish Angora | 1 (0.2) | 12.0 | 95.8 | 4.2 (0.59-30.03) | 0.589 |

Missings in breed: 68

Table S4: Incidence rates and confidence interval (95%), according with tumor topography and animal species.

|  | **IR (95% CI)** | | | | | |
| --- | --- | --- | --- | --- | --- | --- |
| **Topography** | **Cats** | | | **Dogs** | | |
|  | **All** | **F** | **M** | **All** | **F** | **M** |
| All digestive tumors | 21.0 (19.35-22.73) | 17.0 (14.96-19.13) | 25.7 (23.00-28.46) | 5.9 (5.46-6.40) | 5.7 (5.04-6.38) | 6.1 (5.48-6.82) |
| Anus and Anal Canal | 0.1 (0.00-0.18) | 0.1 (0.00-0.20) | 0.1 (0.00-0.23) | 0.0 (0.01-0.08) | 0.0 (0.00-0.06) | 0.1 (0.00-0.13) |
| Colon | 0.8 (0.46-1.07) | 0.5 (0.13-0.86) | 1.1 (0.54-1.69) | 0.5 (0.25-0.47) | 0.3 (0.18-0.48) | 0.4 (0.24-0.56) |
| Gallbladder | 0.1 (0.00-0.18) | 0.1 (0.00-0.20) | 0.1 (0.00-0.23) | 0.1 (0.01-0.10) | 0.2 (0.00-0.14) | 0.0 (0.00-0.09) |
| Liver | 1.5 (1.10-1.99) | 1.5 (0.86-2.12) | 1.6 (1.00-2.31) | 1.5 (1.30-1.77) | 1.8 (1.48-2.22) | 1.2 (0.96-1.54) |
| Other and ill-defined digestive organs | 7.5 (6.51-8.54) | 5.6 (4.43-6.88) | 9.7 (8.00-11.38) | 1.1 (0.93-1.35) | 0.9 (0.66-1.20) | 1.3 (0.98-1.60) |
| Pancreas | 0.4 (0.21-0.68) | 0.5 (0.13-0.86) | 0.4 (0.08-0.77) | 0.2 (0.13-0.29) | 0.2 (0.08-0.34) | 0.2 (0.09-034) |
| Peritoneum | 0.5 (0.21-0.75) | 0.5 (0.13-0.86) | 0.5 (0.15-0.85) | 0.2 (0.08-0.22) | 0.1 (0.04-0.22) | 0.8 (0.08-0.28) |
| Rectum | 0.2 (0.07-0.39) | 0.2 (0.00-0.46) | 0.2 (0.00-0.54) | 1.1 (0.92-1.34) | 0.8 (0.58-1.08) | 1.4 (1.11-1.73) |
| Small intestine | 7.5 (6.51-8.54) | 5.6 (4.43-6.88) | 9.7 (8.00-11.38) | 1.1 (0.91-1.31) | 0.9 (0.66-1.20) | 1.3 (0.98-1.60) |
| Stomach | 1.5 (1.10-1.99) | 1.1 (0.60-1.59) | 2.1 (1.31-2.92) | 0.5 (0.40-0.68) | 0.5 (0.34-0.76) | 0.5 (0.34-0.73) |

Table S5: Relative risks and confidence interval (95%), according with tumor topography and animal species.

| **Topography** | **RR_(cat/dog)_ (95% CI)** | ***p*-value** |
| --- | --- | --- |
| All digestive tumors | 3.6 (2.83-4.45) | <0.001 |
| Anus and Anal Canal | 1.8 (0.06-54.76) | 0.726 |
| Colon | 2.1 (0.71-6.09) | 0.179 |
| Gallbladder | 1.5 (0.06-39.04) | 0.818 |
| Liver | 1.0 (0.51-1.96) | 1.000 |
| Other and ill-defined digestive organs | 6.6 (4.22-10.41) | <0.001 |
| Pancreas | 2.1 (0.51-8.67) | 0.305 |
| Peritoneum | 3.2 (0.72-14.06) | 0.126 |
| Rectum | 0.2 (0.04-0.98) | <0.001 |
| Small intestine | 6.8 (4.31-10.73) | <0.001 |
| Stomach | 2.9 (1.29-6.38) | <0.001 |

Table S6: Relative risks and confidence interval (95%) male *versus* female, according with tumor topography and animal species.

|  | **Cats** | | **Dogs** |  |
| --- | --- | --- | --- | --- |
| **Topography** | **RR (95% CI)** Male/female | ***p*-value** | **RR (95% CI)** Male/female | ***p*-value** |
| All digestive tumors | 1.5 (1.36-1.68) | <0.001 | 1.1 (0.97-1.20) | 0.090 |
| Anus and Anal Canal | 1.1 (0.00-inf) | 1.000 | 3.0 (0.75-12.00) | 0.317 |
| Colon | 2.4 (1.36-4.05) | 0.127 | 1.2 (0.79-1.89) | 0.411 |
| Gallbladder | 1.1 (0.00-inf) | 1.000 | 0.7 (0.009-4.76) | 0.655 |
| Liver | 1.1 (0.72-1.72) | 0.879 | 0.7 (0.53-0.85) | <0.001 |
| Other and ill-defined digestive organs | 1.7 (1.44-2.05) | <0.001 | 1.4 (1.09-1.77) | <0.001 |
| Pancreas | 0.8 (0.31-2.21) | 0.564 | 1.1 (0.56-1.95) | 0.827 |
| Peritoneum | 1.0 (0.42-2.40) | 0.782 | 1.4 (0.71-2.84) | 0.439 |
| Rectum | 1.2 (0.29-4.60) | 1.000 | 1.7 (1.37-2.16) | <0.001 |
| Small intestine | 1.7 (1.44-2.05) | <0.001 | 1.4 (1.09-1.77) | <0.001 |
| Stomach | 2.0 (1.33-2.88) | 0.093 | 1.0 (0.67-1.43) | 0.893 |

Table S7: Relative risks and confidence interval (95%) by morphology and species (cat/dog).

| **Morphology** | **RR_(cat/dog)_ (95% CI)** | ***p*-value** |
| --- | --- | --- |
| Adenocarcinoma | 2.0 (1.15-3.40) | <0.001 |
| Adenoma | 0.6 (0.22-1.55) | 0.280 |
| Carcinoma | 0.8 (0.30-2.30) | 0.715 |
| GIST | 0.7 (0.04-15.31) | 0.842 |
| Hemangioma | 3.7 (0.07-185.17) | 0.515 |
| Hemangiosarcoma | 0.8 (0.16-4.13) | 0.793 |
| Insulinoma | 1.8 (0.06-54.76) | 0.726 |
| Leiomyosarcoma | 0.5 (0.08-3.42) | 0.500 |
| Lymphoma | 16.4 (10.46-25.71) | <0.001 |
| Mast cell tumor | 3.2 (0.42-24.45) | 0.259 |
| Neoplasm, malignant | 3.2 (0.36-27.88) | 0.303 |
| Osteosarcoma | 2.8 (0.14-55.01) | 0.507 |
| Round cell tumor | 2.5 (0.46-13.92) | 0.282 |
| Sarcoma | 1.5 (0.40-5.84) | 0.542 |

Table S8: Incidence rates and confidence interval (95%) by morphology, species and sex.

|  | **IR (95% CI)** | | | |
| --- | --- | --- | --- | --- |
| **Morphology** | **Cats** | | **Dogs** | |
|  | **F** | **M** | **F** | **M** |
| Lymphoma | 11.1 (9.44-12.80) | 19.0 (16.63-21.37) | 0.9 (0.67-1.21) | 0.9 (0.61-1.11) |
| Adenocarcinoma | 2.5 (1.72-3.32) | 3.2 (2.25-4.21) | 1.4 (1.07-1.73) | 1.5 (1.16-1.81) |
| Adenoma | 0.5 (0.16-0.90) | 0.9 (0.35-1.35) | 1.1 (0.83-1.41) | 1.2 (0.89-1.48) |
| Carcinoma | 0.9 (0.44-1.41) | 0.3 (0.01-0.61) | 0.7 (0.50-0.98) | 0.8 (0.57-1.05) |
| Sarcoma | 0.5 (0.16-0.90) | 0.3 (0.01-0.61) | 0.3 (0.15-0.45) | 0.3 (0.12-0.40) |
| Round cell tumor | 0.2 (0.00-0.42) | 0.5 (0.09-0.83) | 0.1 (0.02-0.22) | 0.1 (0.03-0.23) |
| Hemangiosarcoma | 0.1 (0.00-0.32) | 0.9 (0.05-0.72) | 0.4 (0.19-0.53) | 0.3 (0.12-0.40) |
| Mast cell tumor | 0.2 (0.00-0.42) | 0.3 (0.01-0.61) | 0.1 (0.01-0.19) | 0.1 (0.00-0.12) |
| Neoplasm, malignant | 0.1 (0.00-0.32) | 0.3 (0.01-0.61) | 0.0 (0.00-0.06) | 0.1 (0.02-0.20) |
| Leiomyosarcoma | 0.2 (0.00-0.42) | 0.2 (0.00-0.37) | 0.3 (0.12-0.40) | 0.4 (0.24-0.59) |
| Osteosarcoma | 0.1 (0.00-0.20) | 0.2 (0.00-0.37) | 0.0 (0.00-0.10) | 0.0 (0.00-0.09) |
| Mesothelioma | 0.1 (0.00-0.32) | 0.1 (0.00-0.23) | - | - |
| GIST | 0.1 (0.00-0.20) | 0.1 (0.00-0.23) | 0.1 (0.00-0.16) | 0.1 (0.02-0.20) |
| Insulinoma | 0.1 (0.00-0.32) | NA | 0.0 (0.00-0.06) | 0.1 (0.00-0.12) |
| Hemangioma | 0.1 (0.00-0.32) | NA | 0.0 (0.00-0.06) | 0.0 (0.00-0.06) |
| Malignant tumor | NA | 0.1 (0.00-0.23) | - | - |
| Leiomyoma | - | - | 0.2 (0.05-0.27) | 0.2 (0.07-0.30) |
| Plasmacytoma, extramedullary | - | - | NA | 0.1 (0.00-0.12) |
| Myxosarcoma | - | - | NA | 0.0 (0.00-0.09) |
| Liposarcoma | - | - | NA | 0.0 (0.00-0.09) |
| Papilloma | - | - | 0.0 (0.00-0.06) | NA |
| Plasma cell myeloma | - | - | NA | 0.0 (0.00-0.06) |

Table S9: Relative risks and confidence interval (95%) by morphology and species (male/female).

|  | **Cats** | | **Dogs** | |
| --- | --- | --- | --- | --- |
| **Morphology** | **RR_(m/f)_ (95% CI)** | ***p*-value** | **RR_(m/f)_ (95% CI)** | ***p*-value** |
| Adenocarcinoma | 1.3 (0.83-1.99) | 0.655 | 1.1 (0.77-1.46) | 0.461 |
| Adenoma | 1.6 (0.65-3.99) | 0.491 | 1.1 (0.74-1.51) | 0.521 |
| Carcinoma | 0.3 (0.11-1.01) | <0.001 | 1.1 (0.71-1.70) | 0.502 |
| GIST | 1.1 (0.07-18.27) | 1.000 | 1.2 (0.39-4.87) | 0.527 |
| Hemangioma | NA | NA | 1.0 (0.06-15.99) | 1.000 |
| Hemangiosarcoma | 2.9 (0.57-15.07) | 0.257 | 0.7 (0.36-1.45) | 0.480 |
| Insulinoma | NA | NA | 3.0 (0.31-28.84) | 0.317 |
| Leiomyoma | - | - | 1.2 (0.47-3.01) | 0.637 |
| Leiomyosarcoma | 0.8 (0.13-4.49) | 0.655 | 1.6 (0.79-3.13) | 0.128 |
| Lymphoma | 1.7 (1.40-2.08) | <0.001 | 0.9 (0.61-1.37) | 0.917 |
| Mast cell tumor | 1.6 (0.35-6.93) | 0.705 | 0.6 (0.14-2.51) | 0.480 |
| Mesothelioma | 0.6 (0.06-6.79) | 0.564 | - | - |
| Neoplasm, malignant | 2.4 (0.44-13.02) | 0.414 | 5.5 (0.66-45.69) | 0.059 |
| Osteosarcoma | 2.1 (0.19-23.63) | 0.564 | 1.0 (0.14-7.10) | 1.000 |
| Round cell tumor | 2.3 (0.58-9.20) | 0.317 | 1.1 (0.36-3.22) | 0.782 |
| Sarcoma | 0.6 (0.18-1.94) | 0.248 | 0.9 (0.42-1.80) | 0.853 |
